# Fully automated annotation of mitochondrial genomes using a cluster-based approach with de Bruijn graphs

**DOI:** 10.3389/fgene.2023.1250907

**Published:** 2023-08-10

**Authors:** Lisa Fiedler, Martin Middendorf, Matthias Bernt

**Affiliations:** ^1^ Department of Computer Science, Leipzig University, Leipzig, Germany; ^2^ Helmholtz Centre for Environmental Research—UFZ, Leipzig, Germany

**Keywords:** annotation, gene prediction, mitochondria, genome, mitogenome, Metazoa, de Bruijn graph, clustering

## Abstract

A wide range of scientific fields, such as forensics, anthropology, medicine, and molecular evolution, benefits from the analysis of mitogenomic data. With the development of new sequencing technologies, the amount of mitochondrial sequence data to be analyzed has increased exponentially over the last few years. The accurate annotation of mitochondrial DNA is a prerequisite for any mitogenomic comparative analysis. To sustain with the growth of the available mitochondrial sequence data, highly efficient automatic computational methods are, hence, needed. Automatic annotation methods are typically based on databases that contain information about already annotated (and often pre-curated) mitogenomes of different species. However, the existing approaches have several shortcomings: 1) they do not scale well with the size of the database; 2) they do not allow for a fast (and easy) update of the database; and 3) they can only be applied to a relatively small taxonomic subset of all species. Here, we present a novel approach that does not have any of these aforementioned shortcomings, (1), (2), and (3). The reference database of mitogenomes is represented as a richly annotated de Bruijn graph. To generate gene predictions for a new user-supplied mitogenome, the method utilizes a clustering routine that uses the mapping information of the provided sequence to this graph. The method is implemented in a software package called DeGeCI **(De** Bruijn graph **Ge**ne **C**luster **I**dentification). For a large set of mitogenomes, for which expert-curated annotations are available, DeGeCI generates gene predictions of high conformity. In a comparative evaluation with MITOS2, a state-of-the-art annotation tool for mitochondrial genomes, DeGeCI shows better database scalability while still matching MITOS2 in terms of result quality and providing a fully automated means to update the underlying database. Moreover, unlike MITOS2, DeGeCI can be run in parallel on several processors to make use of modern multi-processor systems.

## 1 Introduction

Mitochondria are spherical organelles found in most eukaryotic cells. Their genome, the mitogenome, differs in various aspects from their nuclear counterpart, which include their size, structure, and composition. In Metazoa, the mitogenome is commonly organized as a double-stranded circular DNA molecule with an average length of approximately 16, 500 nt and a small core set of 37 genes, comprised of 13 protein-coding genes, 22 tRNAs, two rRNAs, and one non-coding region, which contains most of the regulatory elements (Wolstenholme, 1992). Although the gene content is generally well conserved, the gene arrangement varies greatly among animal mitogenomes. This renders them an attractive target for a variety of comparative analyses, such as phylogenetic reconstruction or genome rearrangement studies. To facilitate such analyses to be performed systematically on a large scale, automated, standardized annotation of the mitogenome is an indispensable prerequisite.

The widest selection of publicly available mitochondrial genome data can be found in the GenBank (Benson et al., 2000) and RefSeq (Pruitt et al., 2007) databases. GenBank offers access to original sequence data, whereas RefSeq provides a non-redundant expert-curated collection of original GenBank entries. Several databases and tool sets have been built on these data repositories to generate (*de novo*) annotations for user-supplied sequence data, e.g., DOGMA (Wyman et al., 2004), MOSAS (Sheffield et al., 2010), MitoFish (Iwasaki et al., 2013), and MITOS (Bernt et al., 2013). These approaches identify genes using either the sequence similarity search against sequence databases, containing gene sequences of published mitogenomes, or search with curated (hidden Markov/covariance) gene models. All the aforementioned approaches identify protein-coding genes using BLASTX and/or BLASTN searches against an internal database. DOGMA, MOSAS, and MitoFish further apply this technique to rRNA gene detection, whereas MITOS uses Infernal (Eddy, 2002; Nawrocki et al., 2009) and covariance models for mitochondrial rRNAs to serve this purpose. For tRNA annotation, MITOS uses covariance models (Eddy and Durbin, 1994), DOGMA employs COVE, MOSAS applies ARWEN and tRNAscan-SE (Lowe and Eddy, 1997), and MitoFish makes use of MiTFi. Meanwhile, an updated and improved version, MITOS2, was developed, which is based on a more current RefSeq release and allows us to search for protein-coding genes with profile hidden Markov models (HMMs) (Donath et al., 2019). One drawback of DOGMA and MOSAS is that they require some manual improvements on the result set. MOSAS’s restriction to insects and MitoFish’s restriction to fishes limit their scope of application. The fast-growing amount of available mitogenomes creates two problems for all of the aforementioned approaches: 1) the runtime for the sequence similarity search increases approximately linearly with the database size and 2) the necessary curation of gene models impedes automatic updates that allow the inclusion of new sequence data that becomes available over time.

de Bruijn graphs (Bruijn, 1946; Good, 1946) are an important data structure for compact sequence data representation. To this end, sequences are decomposed into small segments, the so-called *k*-mers, which form the vertices of this graph. Two vertices are connected if the suffix of length *k* − 1 of the first vertex is equal to the prefix of length *k* − 1 of the second vertex. In the field of bioinformatics, de Bruijn graphs have often been used for DNA fragment assembly, such as in Pevzner et al. (2001), Pevzner et al. (2004), and Zerbino and Birney (2008). The latter employs a modified de Bruijn graph, the A-Bruijn graph, which can also be used for repeat classifications. Another variant is the manifold de Bruijn graph (Lin and Pevzner, 2014), which allows using (*k* + 1)-mers of variable lengths, choosing larger values for high-coverage regions and smaller values for low-coverage regions. However, the focus of these applications has been on nuclear genomes. Their huge sequence length, as opposed to mitochondrial genomes, explains the emergence of vastly compressed storage structures proposed in the literature to keep the required amount of memory as small as possible. One such structure is introduced by Bowe et al. (2012). Almodaresi et al. (2017) extended this approach by additionally allowing us to store a single property, the “color,” per edge. For many applications, such as variant detection, the approach is sufficient where keeping track of the identity of each of the contributing sequences is the only focus. However, if several properties need to be considered, this storage structure cannot be used. Another downside, which also applies to A-Bruijn and manifold de Bruijn graphs, is that they are all generated based on a fixed set of input genomes. When additional sequences need to be embedded or some contained sequences need to be removed, the entire graph must be reconstructed, which is already, for a moderate amount of genomes and/or long sequence lengths, a time-consuming task.

This work presents DeGeCI (**De** Bruijn graph **Ge**ne **C**luster **I**dentification), a new method for the efficient automatic gene detection of mitochondrial genomes. This method uses a collection of mitogenomes, whose sequence data are represented as a richly annotated **M**itochondrial **D**e **B**ruijn **G**raph (*MDBG*). To annotate an input sequence *r*_in_, a subgraph $MDBG\left[K_{r_{in}}\right]$ induced by all (*k* + 1)-mers of *r*_in_ is initially constructed. Unmapped sequence portions result in disconnected components in this subgraph, which are bridged in the following step. To this end, alternative trails in the *MDBG*, exhibiting a high sequence similarity to the respective unmapped subsequences of *r*_in_, are identified and added to $MDBG\left[K_{r_{in}}\right]$ (Section 2.2.2). Using a clustering approach, DeGeCI aggregates annotations of the subgraph to obtain gene predictions for the input sequence (Section 2.2.3). In this study, we use a comprehensive set of all 8,015 mitogenomes contained in RefSeq 89, covering all major metazoan taxonomic groups, to construct the database graph. Gene predictions are computed for a large and taxonomically representative sample of mitogenomes and are compared to existing expert-curated annotations and MITOS2 (Section 4.3).

## 2 Methods

### 2.1 Graph structure

Given a string (i.e., a sequence of characters), a *k*-mer is a substring of length *k*. A string can be disassembled into all of its (*k* + 1)-mers by sliding a window of length (*k* + 1) over the string while retaining duplicates. A genome *r* is a string composed of nucleotides A, C, T, and G, where circular genomes are linearized by “cutting” the genome at an arbitrary but fixed location.

**FIGURE 1:**
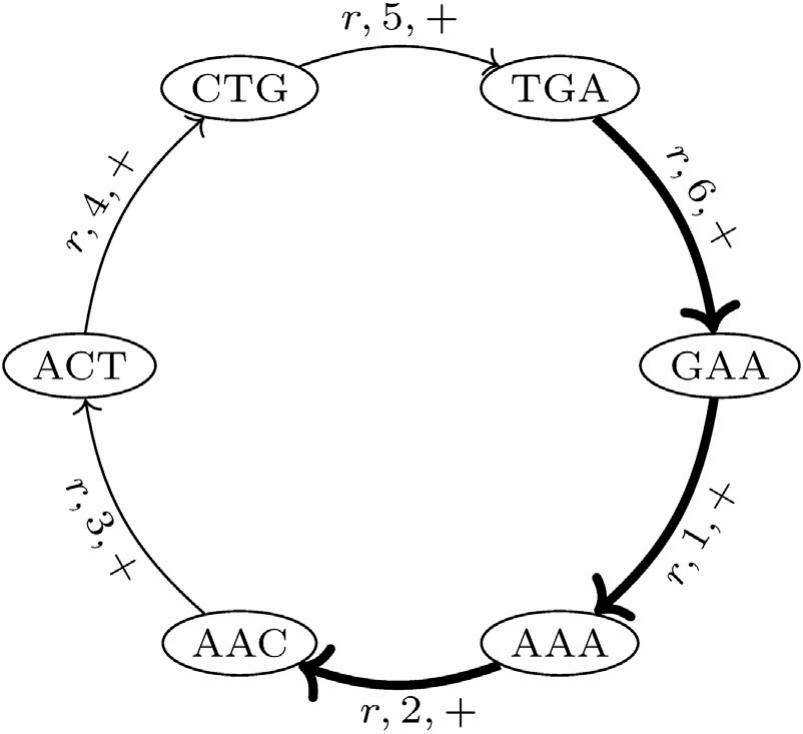
de Bruijn graph of a circular genome *r* with the sequence ACTGAA for *k* = 3 and positive strand *σ* = +. The SGT (3, 2, *r*, +) is exemplarily shown. The corresponding edges in the graph are highlighted in bold.

In the proposed de Bruijn graph, *MDBG* (*V*, *E*), over a set of genomes *G* with the vertex set *V* and edge set *E*, each (*k* + 1)-mer *x*_1_ *x*_2_ … *x*_*k*+1_ of every genome in *G* leads to two vertices *v* and *v*′ in *V*, representing the *k*-prefix *x*_1_ *x*_2_ … *x*_*k*_ and *k*-suffix *x*_2_ … *x*_*k*+1_, respectively. These are connected by directed edges (*v* and *v*′), which represent the (*k* + 1)-mer itself. For circular genomes, the (*k* + 1)-mers that connect both sides of the linearized string representation are also included. Thus, while each linear genome of length |*r*| contributes |*r*| − *k* many (*k* + 1)-mers, a circular genome of this length contributes |*r*| many (*k* + 1)-mers to the graph. The complementary DNA strand (negative strand), with an opposite reading direction, is taken into account by adding the reverse complement of each (*k* + 1)-mer to *MDBG*. Each edge (*v* and *v*′) is annotated with a label *r*, denoting the genome from which this edge originates, the strandedness *σ* ∈ { +, − }, and the position *p* (with respect to the positive strand) of nucleotide *x*_*k*+1_ of (*v* and *v*′) in genome *r*. This allows for an unambiguous reconstruction of each genome in *G* from *MDBG* (*V*, *E*). It should be noted that each (*k* + 1)-mer that is contained in multiple different genomes or is contained multiple times in the same genome (i.e., due to repeats) results in a pair of vertices that is connected by multiple edges, the so-called parallel edges. The *MDBG* is thus a multigraph. Figure 1 illustrates an example of the de Bruijn graph of a circular genome *r* with the sequence ACTGAA for *k* = 3 on the positive strand.

A trail in a graph is a sequence of distinct edges that joins a sequence of vertices. Let (*i*, *j*, *r*, *σ*) be the trails in *MDBG*, denoting a *single genome trail* (SGT), which is composed of edges corresponding to the subsequence from the position *i* to *j* in the genome *r* located on the strand *σ*. For a circular genome, *i* >*j*, if the associated subsequence extends over the string boundary of the linearized genome representation. In the de Bruijn graph depicted in Figure 1, one such SGT is exemplarily highlighted.

### 2.2 Workflow

**FIGURE 2:**
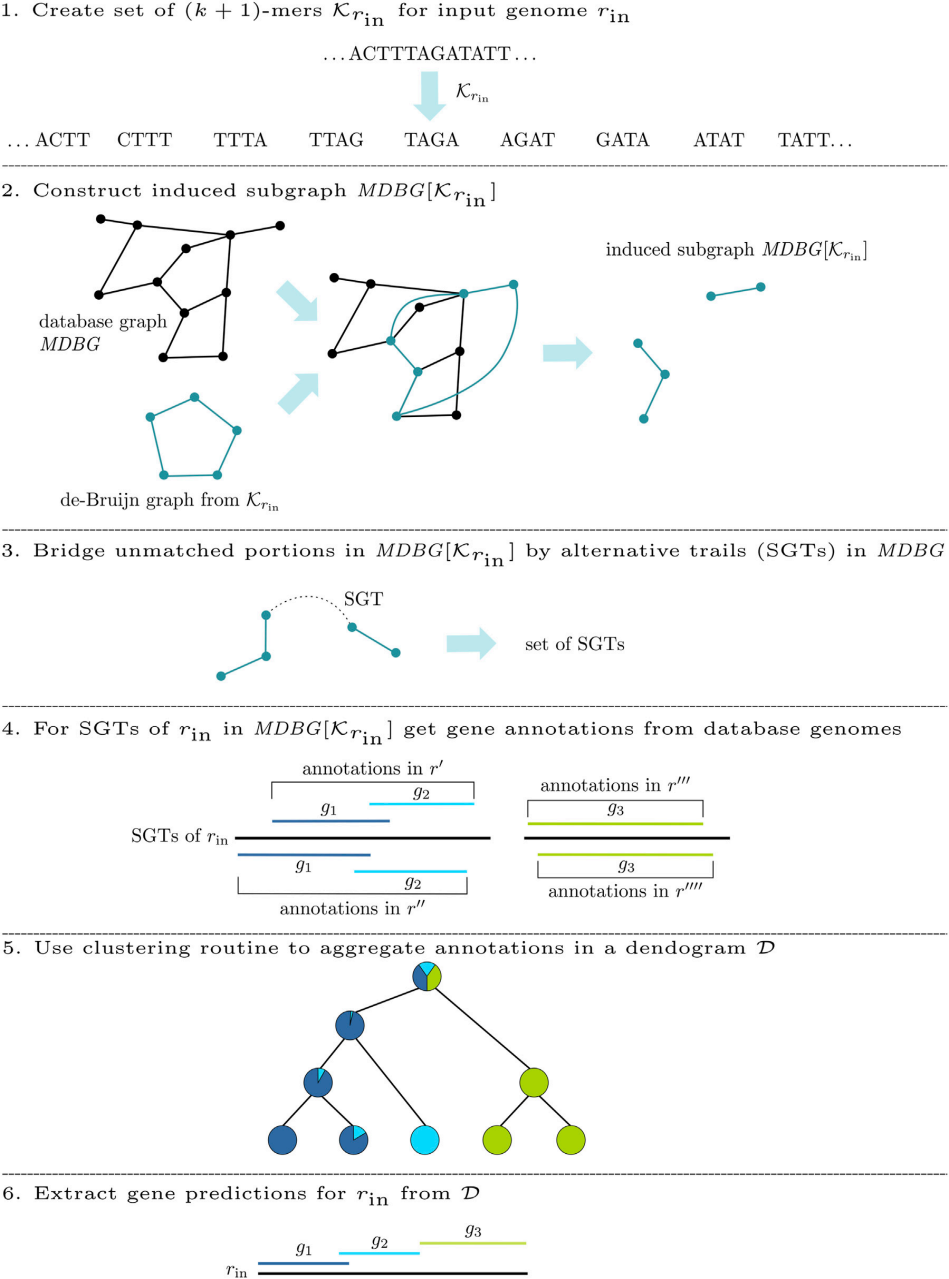
DeGeCI workflow for the *de novo* annotation of an input genome *r*_in_.

For the *de novo* annotation of an input genome *r*_in_, DeGeCI requires only its nucleotide sequence. The DeGeCI pipeline consists of six major stages, which are summarized in Figure 2. The following sections present the individual steps involved.

#### 2.2.1 Subgraph construction

Initially, DeGeCI generates the set $K_{r_{in}}$ of all (*k* + 1)-mers of the input genome *r*_in_. Next, the subgraph $MDBG\left[K_{r_{in}}\right]$ , which is induced by all (*k* + 1)-mers in the database graph *MDBG* that are also contained in $K_{r_{in}}$ , is constructed. For each such *matching* (*k* + 1)-mer in $K_{r_{in}}$ , an edge is added to $MDBG\left[K_{r_{in}}\right]$ and labeled with *r*_in_, the related sequence position *p*, and the strand $\sigma_{r_{in}}$ of *r*_in_. Thus, there are at least two edges between each pair of vertices in the subgraph: one of *r*_in_ and one of a database genome *r*.

#### 2.2.2 Connected component bridging

Even if dense taxon sampling is provided in the database graph, input species with a poorly conserved gene content can lead to (*k* + 1)-mers in $K_{r_{in}}$ that do not map to any edge in the *MDBG* for a reasonable value of *k* (see Section 4.2.1). These non-matching (*k* + 1)-mers decompose the genome’s continuous sequence of (*k* + 1)-mers in the consecutive blocks of matching (*k* + 1)-mers. Consequently, the subgraph $MDBG\left[K_{r_{in}}\right]$ is composed of smaller subgraphs, each of which is induced by one such subsequence block of (*k* + 1)-mers. Going forward, these subgraphs will be called connected components (CCs). Thus, two vertices are part of the same CC if there is an SGT of the input genome *r*_in_ that connects them.

While two CCs of $MDBG\left[K_{r_{in}}\right]$ are not connected by SGTs of *r*_in_, there may be SGTs of other genomes in the database graph *MDBG*, which connect them. More precisely, there might be an SGT *t*_1_ of some genome *r* ∈ *G* in one CC *C*_*I*_ and another SGT *t*_2_ of this genome in another CC *C*_*T*_ in $MDBG\left[K_{r_{in}}\right]$ , which might be connected by a third SGT *t*_3_ of *r* in *MDBG*. Such *connecting* trails *t*_3_ between two *seeding* SGTs, *t*_1_ and *t*_2_, could constitute suitable alternative trails for the unmapped sequence segments in $MDBG\left[K_{r_{in}}\right]$ , thereby bridging pairs of CCs *C*_*I*_ and *C*_*T*_.

**FIGURE 3:**
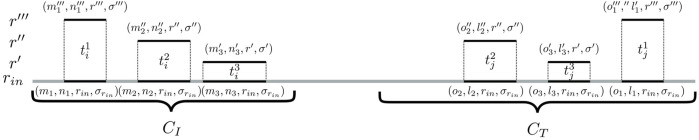
Identification of bridging trails for components *C*_*I*_ and *C*_*T*_. In this example, there are three pairs of seeding SGTs $\left(t_{i}^{1},t_{j}^{1}\right),\left(t_{i}^{2},t_{j}^{2}\right)$ , and $\left(t_{i}^{3},t_{j}^{3}\right)$ of genomes *r*′′′, *r*″, and *r*′, respectively. The corresponding bridging trails are $\left(n_{1}^{\prime \prime \prime }+1,o_{1}^{\prime \prime \prime }-1,r\prime \prime \prime ,\sigma^{\prime \prime \prime }\right),\left(n_{2}\prime \prime +1,o_{2}\prime \prime -1,r\prime \prime ,\sigma \prime \prime \right)$ , and $\left(n_{3}^{\prime }+1,o_{3}^{\prime }-1,r^{\prime },\sigma^{\prime }\right)$ .

To identify such connecting trails, an algorithm called cc-bridging was developed. The pseudocode is given in Algorithm 1. For each CC *C*_*I*_, induced by subsequence *s*_*I*_, bridging is initially attempted with CC *C*_*T*_, induced by subsequence *s*_*T*_, which among all inducing subsequences of CCs in $MDBG\left[K_{r_{in}}\right]$ is (in the reading direction) located closest to *s*_*I*_ in the input genome (line 6). This serves to retain sequence locality. For this pair of CCs, the algorithm identifies bridging trails between suitable pairs of seeding trails (line 16). The individual steps of this bridging routine are outlined in Section 2.2.2.1. To prevent a large number of mostly unsuitable seeding trails being validated, only a small portion of both CCs is considered at first. The portions are only extended if no appropriate bridging trails can be identified within them. This is controlled by a parameter $N∋g\geq g_{0}=2$ , which might get adapted during program execution (line 20). If at least one of the two CCs was already searched completely, *C*_*T*_ is updated to the next closest CC (line 23) and the aforementioned routine starts over again. This is repeated until valid bridging trails are found or if the distance $\delta_{s_{I},s_{T}}$ between the inducing subsequences is more than twice as large as either of their lengths (line 14). The latter is a rough filter for speed-up purposes, with the reasoning that the larger the distance between the inducing subsequences and the smaller their lengths, the less likely it is to find suitable bridging trails between their CCs. Figure 3 visualizes this setup.

Algorithm 1. CC-BRIDGING.

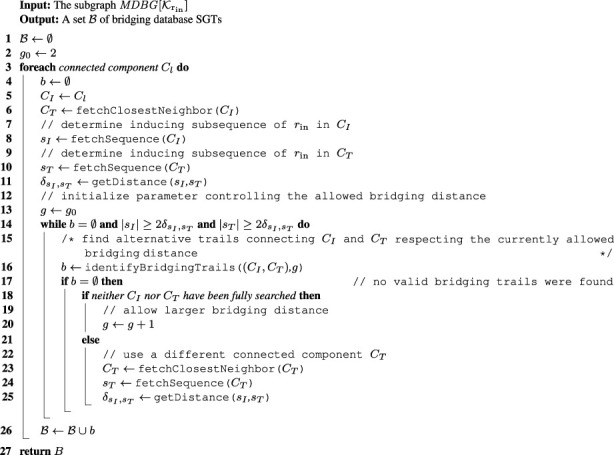

##### 2.2.2.1 Validation of seeding trails

The validation of the seeding trails for two CCs, *C*_*I*_ and *C*_*T*_ (line 16 in Algorithm 1), consists of the following two steps:

Step 1: Identification of bridging candidates. The method searches for seeding SGTs (*m*′, *n*′, *r*, *σ*) ∈ *C*_*I*_ and (*o*′, *l*′, *r*, *σ*) ∈ *C*_*T*_ so that there are *mapping* input genome SGTs $\left(m,n,r_{in},\sigma_{r_{in}}\right)\in C_{I}$ and $\left(o,l,r_{in},\sigma_{r_{in}}\right)\in C_{T}$ , and it is not possible to further extend these mappings. Here, mapping means that all (*k* + 1)-mers of the respective database SGT coincide with those of the input SGT. For each such pair of seeding SGTs, a possible bridging trail is given by (*n*′ + 1, *o*′ − 1, *r*, *σ*).

The two input genome SGTs should be as close as possible to each other to ensure sequence locality. The smallest possible distance between them is $\delta_{s_{I},s_{T}}$ since the inducing subsequences *s*_*I*_ and *s*_*T*_ are separated by this value. Here, we accept SGTs with a distance of up to $g\delta_{s_{I},s_{T}}$ , where *g* is the integer parameter introduced in the previous section. This allows us to adapt the cutoff distance in consecutive method iterations.

**FIGURE 4:**
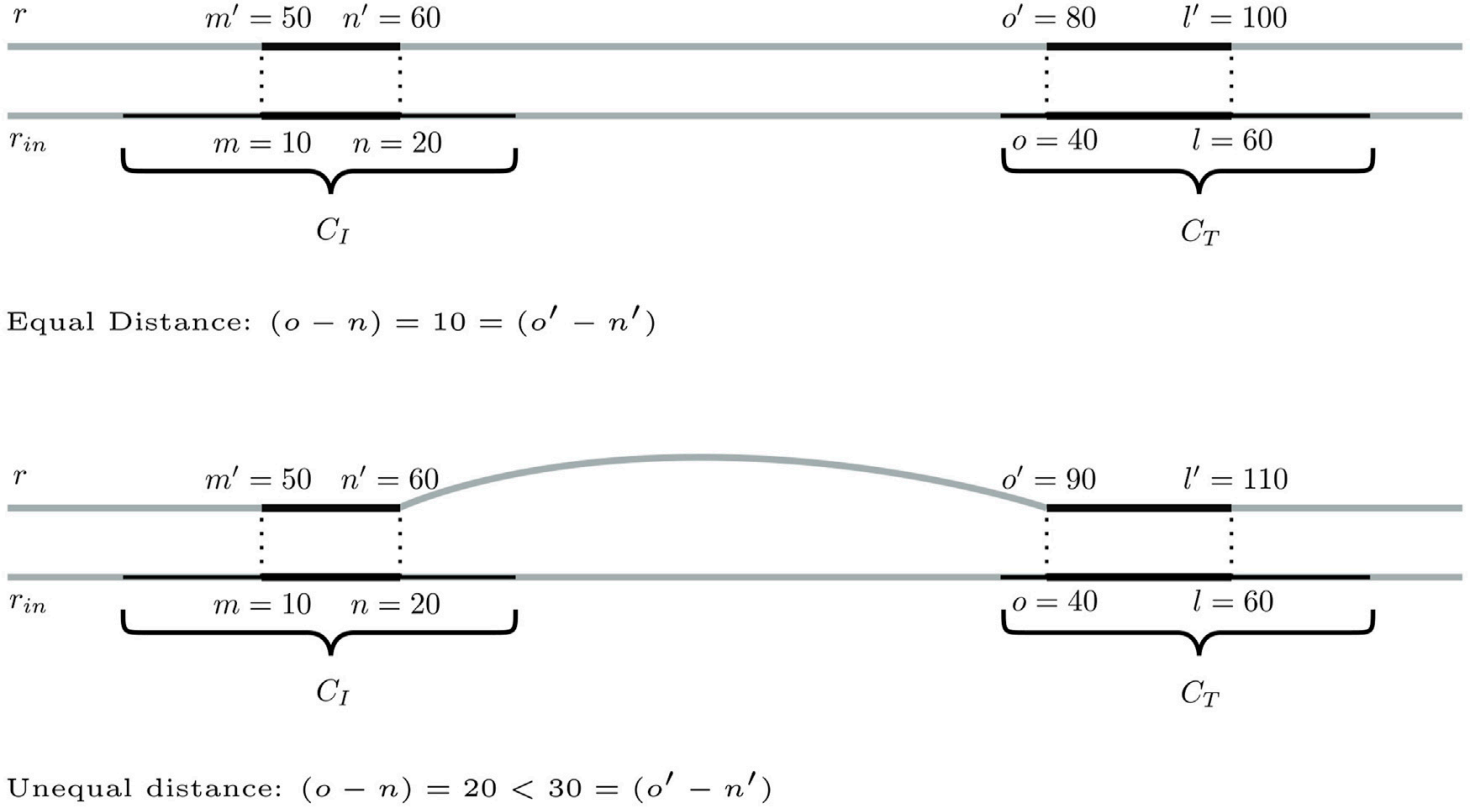
Two scenarios for a pair of seeding SGTs of database genome *r* in CCs *C*_*I*_ and *C*_*T*_, for which a distance of $\delta_{s_{I},s_{T}}=12$ shall be assumed. The distance between the input genome SGTs is 20 in both cases. This means that even with the initial setting of *g* = *g*_0_ = 2, these trails are sufficiently close to one another, i.e., closer than $g_{0}\delta_{s_{I},s_{T}}=24$ . Thus, only the relative distance determines whether to retain SGTs. In the top figure, database and input genome SGTs have a relative distance of zero. The seeding SGTs will, hence, be considered further. In the bottom figure, the relative distance of both SGT pairs is |20 − 30|/20 = 0.5, which exceeds the upper bound of 0.2. These trails are, hence, rejected.

Another important aspect to consider is the sequence similarity of the bridging trail with the unmapped subsequence of *r*_in_. By requesting a small relative distance |(*o* − *n*) − (*o*′ − *n*′)|/(*o* − *n*) of both SGT pairs, the likelihood of a high sequence similarity can be increased without actually evaluating it at this point of the algorithm. In this contribution, we impose an upper bound of 0.2. Examples illustrating the application of the aforementioned two criteria are depicted in Figure 4.

If there is more than one pair of seeding SGTs of the same database genome *r*, the validation routine only retains the pair that is the closest, together with respect to the mapping input genome SGTs, and, in case of a tie, has a lower relative distance.

The algorithm operates on the positions of SGT mappings. Two SGTs of the same genome that correspond to the same sequence, i.e., are a repeat in that genome, have different positions because they are located in different regions of the genome. The algorithm thus treats them exactly the same as any other SGT. Therefore, repeats are not a special case for the algorithm.

Step 2: Pairwise sequence alignments. For each of the remaining SGT pairs, the corresponding bridging trails are examined for sequence similarity to the input genome. To this end, the algorithm conducts local pairwise sequence alignments with affine gap costs (cf. Supplementary Material: Section 5 for details) between the unmapped input sequence segment and sequences that correspond to the bridging trails. Alignments are accepted if they have an E-value of at most 10^–3^.

#### 2.2.3 Gene annotation

The basis for the annotation of the input genome *r*_in_ is a collection of gene annotations A of the database genomes. An element (*n*, *m*, *r*, *σ*_*g*_, *g*) of A denotes that gene *g* is annotated on the strand *σ*_*g*_ from position *n* to *m* in the genome *r* ∈ *G*. A special gene *g*_0_ is used to label regions where no gene is annotated. This serves to avoid a bias toward a small number of random predictions in a later stage of the method. Moreover, each copy or fragment of the same gene gets a different label *g* and, hence, results in a new annotation in A .

**FIGURE 5:**
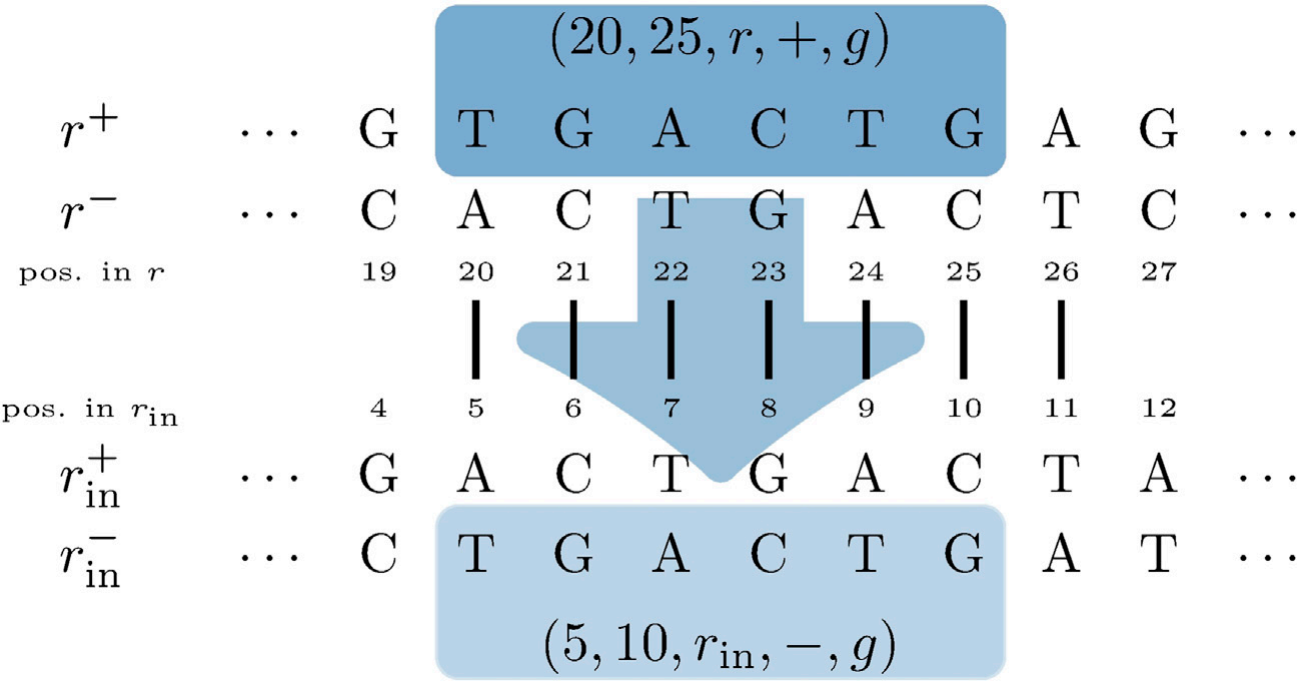
Input genome SGT $\left(i,j,r_{in},\sigma_{r_{in}}\right)=\left(5,11,r_{in},+\right)$ mapping to a database SGT (*i*′, *j*′, *r*, *σ*) =(20,26, *r*, −) (vertical black lines). The example assumes an annotation (*n*, *m*, *r*, *σ*_*g*_, *g*) = (1, 25, *r*, + ,*g*) in A . In other words, the encoding sequence for *g* is located on the opposing strand of the mapping database SGT. This leads to a sub-annotation (*n*′, *m*′, *r*, *σ*_*g*_, *g*) = (20, 25, *r*, + ,*g*) (dark blue box on the top), resulting in an annotation of gene *g* from position 5 to 10 in *r*_in_ on the negative strand (light blue box at the bottom), since $\sigma_{r_{in}}=+\neq -=\sigma$ .

For each pair of mapping SGTs $\left(i,j,r_{in},\sigma_{r_{in}}\right)$ and (*i*′, *j*′, *r*, *σ*) that have been obtained from the subgraph $MDBG\left[K_{r_{in}}\right]$ or the bridging routine in the previous steps, the position overlap of [*i*′, *j*′] with all annotations $\left(n,m,r,\sigma_{g},g\right)\in A$ is evaluated. This results in sub-annotations (*n*′, *m*′, *r*, *σ*_*g*_, *g*), where [*n*′, *m*′] = [*i*′, *j*′] ∩ [*n*, *m*], yielding an annotation for *r*_in_ from *i* + (*n*′ − *i*′) to *j* − (*j*′ − *m*′), which is located on the strand *σ*_*g*_ if $\sigma =\sigma_{r_{in}}$ and otherwise on the opposing strand. This is because $\sigma \neq \sigma_{r_{in}}$ means that the mapping database SGT and input SGT reside on different strands, indicating that the encoding sequences of both genes are located on opposite strands. Figure 5 illustrates an example of the aforementioned annotation process for one database and input genome SGT mapping.

An annotation in A generally results in multiple smaller sub-annotations for *r*_in_, which get separated by small sequence dissimilarities between the input genome and the database genome. If two such fragments originate from the same annotation in A and are separated by a distance smaller than the shortest gene that is typically present in the class of species under consideration (e.g., approximately 70 nt for metazoan mitogenomes), it can be assumed that the missing region in between also encodes the same gene. Thus, DeGeCI iteratively replaces such fragments with a single longer annotation of the respective gene.

It is to be expected that flawed annotations in A and/or random (*k* + 1)-mer matches cause some incorrect gene predictions for *r*_in_. DeGeCI thus utilizes aggregations of annotations, so that the predominant and, therefore, more likely predictions can be identified. To this end, the following clustering routine clusterG was developed. The goal of the routine is to identify as large a region in the input genome as possible where the same few genes are most likely to occur, thereby filtering out noise contained in database gene annotations. Starting from the database gene annotations for each position of *r*_in_, the routine builds a hierarchy of gene prediction clusters by greedily merging the annotations of neighboring positions in a bottom-up fashion. The frequency with which annotations for specific genes occur at a given position determines the likelihood of these genes being correct predictions for that position. Moreover, positions with a similar gene distribution are likely to belong together. This distribution is thus used as a quality measure for identifying the merge quality of two neighboring clusters. For merged clusters, the gene annotations that occur in only one of the clusters are removed to derive an increasingly consistent prediction for combined position ranges of merged clusters. Lastly, gene annotations with a high probability are retrieved from different levels of the generated hierarchy of clusters. The detailed steps are described in the following sections.

##### 2.2.3.1 Aggregating gene predictions

The pseudocode of clusterG is given in Algorithm 2. The algorithm is performed individually with gene predictions $A_{r_{in}}^{+}$ for the positive strand *σ* = + and gene predictions $A_{r_{in}}^{-}$ for the negative strand *σ* = − of the input genome *r*_in_.

Algorithm 2. CLUSTERG.

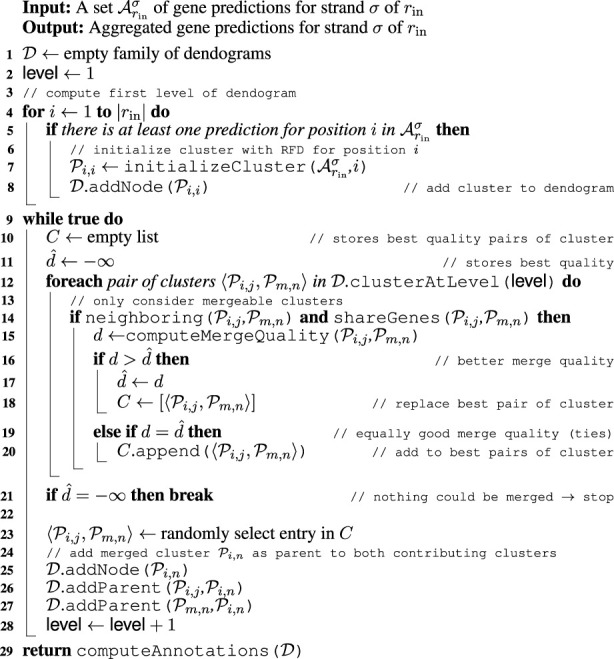

Initially, every position *i* of *r*_in_ constitutes a cluster $P_{i,i}$ . Each such cluster is initialized with a relative frequency distribution (RFD) using gene predictions $A_{r_{in}}^{\sigma }$ (line 7). This RFD is defined as

$$p_{i,i}\left(g\right)=\frac{\omega_{g}S_{i,i}^{g}}{\sum_{\tilde{g}}\omega_{\tilde{g}}S_{i,i}^{\tilde{g}}}≕\frac{{\hat{S}}_{i,i}^{g}}{\sum_{\tilde{g}}{\hat{S}}_{i,i}^{\tilde{g}}},$$ (1)

where $S_{i,i}^{g}$ is the sum of the lengths of gene *g* predictions for the strand *σ* that include position *i* and *ω*_*g*_ is the reciprocal of the length of the gene on average. This assigns higher probabilities to longer and, hence, more trustworthy predictions while preventing bias toward long genes. To warrant a certain degree of reliability, genes are only included in distribution 1 if there are at least two predictions of different database genomes. Moreover, clusters without predictions are removed (line 5).

Once the clusters are initialized, the iterative merging begins (line 9). At each iteration point, the pair of clusters with the highest quality (defined in the following steps) among all mergeable clusters is identified (lines 15–20). Two clusters $P_{i,j}$ and $P_{m,n}$ are considered mergeable if they are neighboring clusters, i.e., *m* − *j* = 1, and their distributions share at least one gene (line 14).

To evaluate the quality of a merge of two clusters $P_{i,j}$ and $P_{m,n}$ , the joined RFD,

$$p_{i,n}\left(g\right)=\frac{\chi_{\left({\hat{S}}_{i,j}^{g}\neq 0\wedge {\hat{S}}_{m,n}^{g}\neq 0\right)}\left({\hat{S}}_{i,j}^{g}+{\hat{S}}_{m,n}^{g}\right)}{\sum_{\tilde{g}}\chi_{\left({\hat{S}}_{i,j}^{\tilde{g}}\neq 0\wedge {\hat{S}}_{m,n}^{\tilde{g}}\neq 0\right)}\left({\hat{S}}_{i,j}^{\tilde{g}}+{\hat{S}}_{m,n}^{\tilde{g}}\right)}$$ (2)

is considered. Here, *χ*_*P*_ equals one if predicate *P* is true and zero otherwise, setting the probability of a gene to zero if it does not appear in both contributing distributions. This is to prevent the annotation of a gene at positions that are not predicted by any of the genomes in the database.

Given such a joined RFD of *n* genes, the smallest number *t* ≤*n* of genes necessary to obtain a cumulated relative frequency of at least 0.95 is identified. Merged clusters with a low value of *t* feature a joined RFD where much weight falls on few genes. This indicates that the two entering clusters can be combined to a consistent prediction, assigning a higher quality to the merge.

At some point, there will most likely be more than one pair of clusters with the smallest value of *t* among all mergeable clusters. In such a case, clusters with a higher maximum score $\max\left\{S^{g}\right\}$ are preferred. If there is a tie, the pair with the largest number of predictions is selected. Finally, in the rare case of remaining ties, the routine selects a pair at random (line 23).

Merging is repeated until no clusters are left that can be joined (line 21). For every merged cluster $P_{i,n}$ , references to the two contributing clusters $P_{i,j}$ (line 26) and $P_{m,n}$ (line 27) are maintained. It should be noted that merging generally stops before all positions are merged into a single cluster. This happens, for example, because there might be neighboring clusters that do not share a single common gene and therefore will never be merged. The outcome is, hence, a family of dendrograms D .

The last step is to retrieve annotations from clusters in D (line 29), described as follows: initially, clusters $P_{i,j}^{⋆}\in D$ are selected where the highest relative frequency max{*p*_*i*,*j*_(*g*)}≔*p*_*i*,*j*_ (*g* ^⋆^) of their RFD is at least 0.7. A high value of *p*_*i*,*j*_ (*g* ^⋆^) means that the vast majority of predictions in $P_{i,j}$ are for *g* ^⋆^, increasing the likelihood that *g* ^⋆^ is the correct annotation for the sequence segment associated with the cluster. Hence, gene *g* ^⋆^ is considered as a putative prediction for this segment.

**FIGURE 6:**
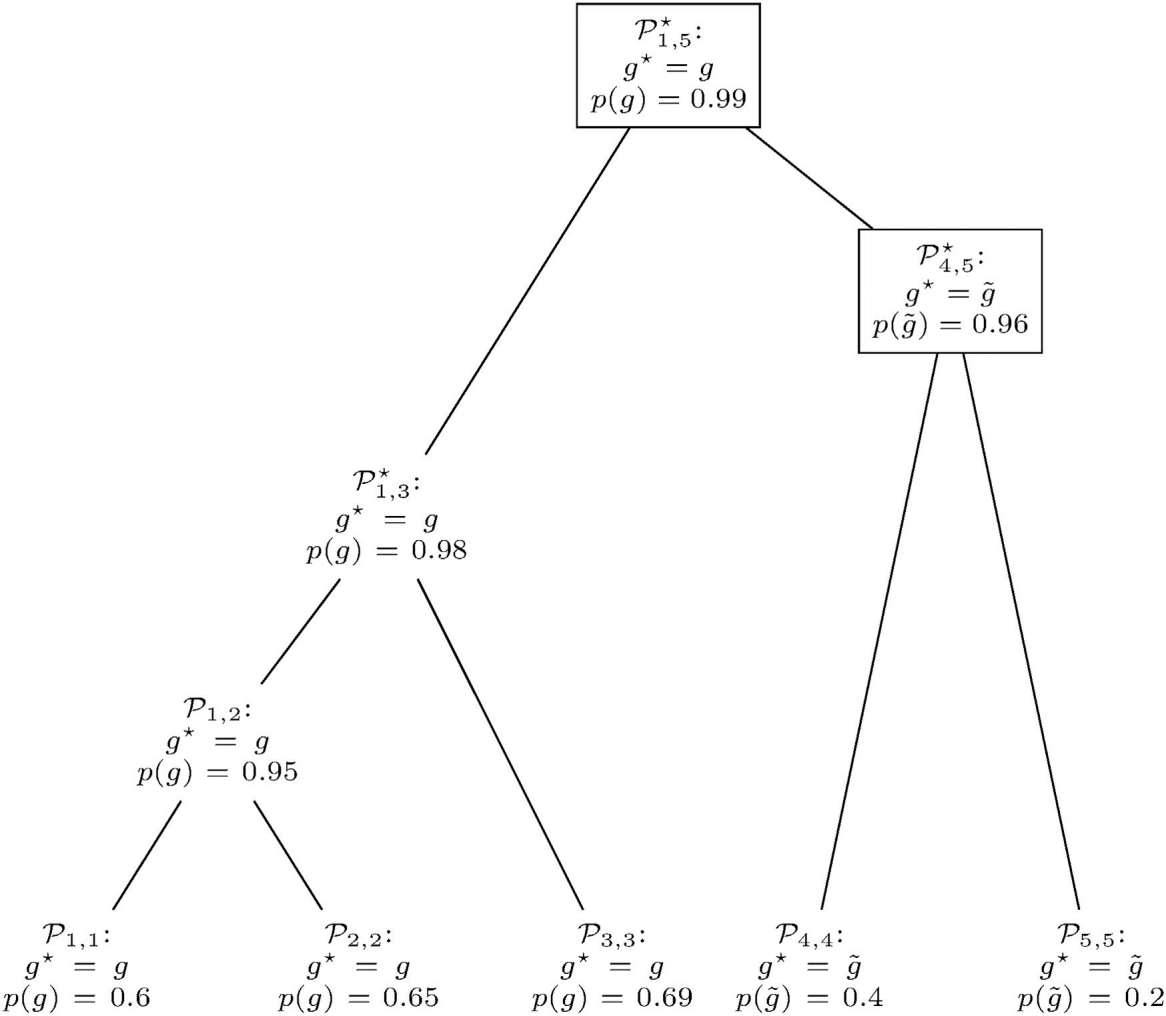
Gene prediction retrieval from the clusters in D . All clusters with maximum relative frequencies of at least 0.7 are marked with an asterisk. Of these, clusters $P_{1,2}^{⋆}$ and $P_{1,3}^{⋆}$ are discarded, since both have the same gene *g* ^⋆^ = *g* as $P_{1,5}^{⋆}$ and [1, 2] ⊂ [1, 3] ⊂ [1, 5]. Thus, only clusters $P_{1,5}$ and $P_{4,5}$ (framed by rectangles) are selected.

Next, clusters $P_{i,j}^{⋆}$ are discarded if there are other clusters $P_{m,n}^{⋆}$ with the same gene *g* ^⋆^ and [*i*, *j*] ⊂ [*m*, *n*]. This is because $P_{i,j}^{⋆}$ does not lead to any information gain regarding the region where *g* ^⋆^ is presumably encoded. However, if *g* ^⋆^ is different in both clusters, $P_{i,j}^{⋆}$ is retained to allow for overlapping predictions. Figure 6 shows an example of this retrieval routine.

For each of the remaining clusters $P_{m,n}^{⋆}$ , gene *g* ^⋆^ is accepted as the prediction from *m* to *n* for the strand *σ*.

##### 2.2.3.2 Handling unannotated regions

We recall that in the initial RFD Eq. 1, each prediction of gene *g* is scaled with weight *ω*_*g*_, which is the reciprocal of the length of such a gene on average. However, there is no reasonable definition of such a length for unannotated regions, i.e., where *g* = *g*_0_. Thus, prior to clustering, positions *p* of *r*_in_, presumably not encoding any actual gene, are identified as follows: there are more predictions with *g* = *g*_0_ than with the total number of predictions of the two (or one if there is only one) best scoring genes *g* ≠ *g*_0_ at *p* or the cumulative relative frequency of these genes falls below 0.8. The rationale behind taking the two best scoring genes into account is that two genes may well overlap, but the precise gene boundaries may vary slightly among different genomes.

### 2.3 Implementation

DeGeCI is available as a free open-source software package^1^. It is implemented in Apache Spark (Veith and de Assuncao, 2019) using its Java API, facilitating parallel program execution. The database graph *MDBG* is stored in an indexed PostgreSQL database (Stonebraker, 1987). PostgreSQL dump files for the database population are available for RefSeq 89 and RefSeq 204^2^.

To initiate the annotation of an input genome, its nucleotide sequence can be provided either as a FASTA file or as a sequence string. The final gene annotation is provided as a bed file.

## 3 Materials

To create the database graph *MDBG*, a comprehensive set of all 8,015 metazoan mitogenomes contained in RefSeq 89 was used. The *k*-mer size was set to *k* = 16, following a careful empirical analysis presented in Section 4.2.1. The curated GenBank files of the RefSeq dataset served as a basis for the gene annotation of SGT mappings. To achieve a consistent nomenclature among all entries, an important prerequisite to derive joint annotation, each GenBank entry was parsed following the guidelines suggested by Boore (2006). The details are compiled in Supplementary Material: Section 2.

To assess the quality of the proposed method, DeGeCI was applied to a sample of 100 genomes of RefSeq 89 (for a complete list, see Supplementary Material: Section 6), for which expert-curated annotations exist. These annotations served as ground truth data, allowing us to assess the accuracy of the produced gene predictions. This sample was drawn by randomly selecting different numbers of genomes from each of the major metazoan groups contained in this RefSeq release. The number of species per group was chosen with respect to the frequency in which they occurred in RefSeq 89 (and, therefore, *MDBG*). This comprises seven Spiralia, 27 Arthropoda, 32 Actinopterygii, four Amphibia, 15 Mammalia, 13 Sauropsida, and two non-bilaterian species. In order to not to bias the gene predictions of an input genome of the sample with its annotation in the database graph, all edges of this genome in the database graph were excluded during the subgraph construction step of DeGeCI.

## 4 Results

This contribution presents a new de Bruijn graph-based method, DeGeCI, for *de novo* gene annotations of mitochondrial genomes. In the following, we compare DeGeCI with MITOS2 (Bernt et al., 2013), a widely used state-of-the-art annotation tool for mitochondrial genomes. Both MITOS2 and DeGeCI are based on an internal database of mitogenomic sequences of the RefSeq database and require only nucleotide sequences as input, allowing for a fair comparison.

While both tools are based on an internal database of mitogenomic sequences, the underlying approaches are essentially different. MITOS2 uses profile hidden Markov models in combination with methods from Donath et al. (2019) and the HMMER software suite (Eddy, 2011) or BLASTX searches (Altschul et al., 1990) for the annotation of protein-coding genes. To detect non-coding RNAs, i.e., tRNAs and rRNAs, MITOS2 uses Infernal (Eddy, 2002; Nawrocki et al., 2009) in the glocal search mode and curated covariance models. Contrarily, DeGeCI is a stand-alone application that relies on no third-party bioinformatics software. Both proteins and RNAs are annotated using the mapping information of the input genome to a database graph, in combination with a subsequent clustering approach.

### 4.1 Benchmarking procedure

To allow for a comparative evaluation with MITOS2, the following definitions are adopted from Bernt et al. (2013). For each DeGeCI/MITOS2 prediction, the corresponding RefSeq prediction is the annotation that has the largest position overlap with the DeGeCI/MITOS2 prediction, given that at least 75% of the DeGeCI/MITOS2 positions are shared with the RefSeq predictions. Each such allocation of a DeGeCI/MITOS2 annotation to a RefSeq annotation is classified as equal if both predict the same gene on the same strand, classified as different if both predict different genes, and classified as having a strand difference if both predict the same gene but on opposing strands. DeGeCI/MITOS2 predictions, where no corresponding RefSeq prediction is found, are classified as false positives (FPs). Analogously, RefSeq predictions without corresponding DeGeCI/MITOS2 predictions are classified as false negatives (FNs).

### 4.2 Parameter settings

For MITOS2, the default parameter setting and appropriate genetic codes were used for each of the 100 considered species. The parameters for DeGeCI were set as follows.

#### 4.2.1 (*k* + 1)-mer size

To determine a suitable value for the (*k* + 1)-mer size of the database graph, the right balance has to be found between a too-small value, leading to a great number of random (*k* + 1)-mer matches, and a too-large value, concealing many sequence similarities among the genomes.

To this end, the following experiment was conducted 100 times for every k∈K≔{6,8,10,12,14,16,18} in turn. First, the multiset (i.e., a set allowing for duplicate entries) $S_{t}$ of all (*k* + 1)-mers of the 8,015 mitochondrial sequences in RefSeq 89 was generated. Next, all sequences were concatenated into a single long sequence, which was subsequently randomly shuffled. From this sequence, a multiset $S_{r}$ of random (*k* + 1)-mers was then constructed. By creating the random set in this way, it features the same nucleotide composition and an almost identical size $|S_{r}|=|S_{t}|-N\left(k+1\right)$ as $S_{t}$ , where *N* is the number of cyclic genomes in RefSeq 89. The average (taken over all 100 experiments) fraction ${\bar{r}}_{\text{hit}}$ of (*k* + 1)-mers in $S_{r}$ that are also contained in $S_{t}$ at least *w*_min_ ≥1 times can then be used to estimate the likelihood of randomly finding (*k* + 1)-mers of the true genome sequences in unrelated sequences of same composition.

**FIGURE 7:**
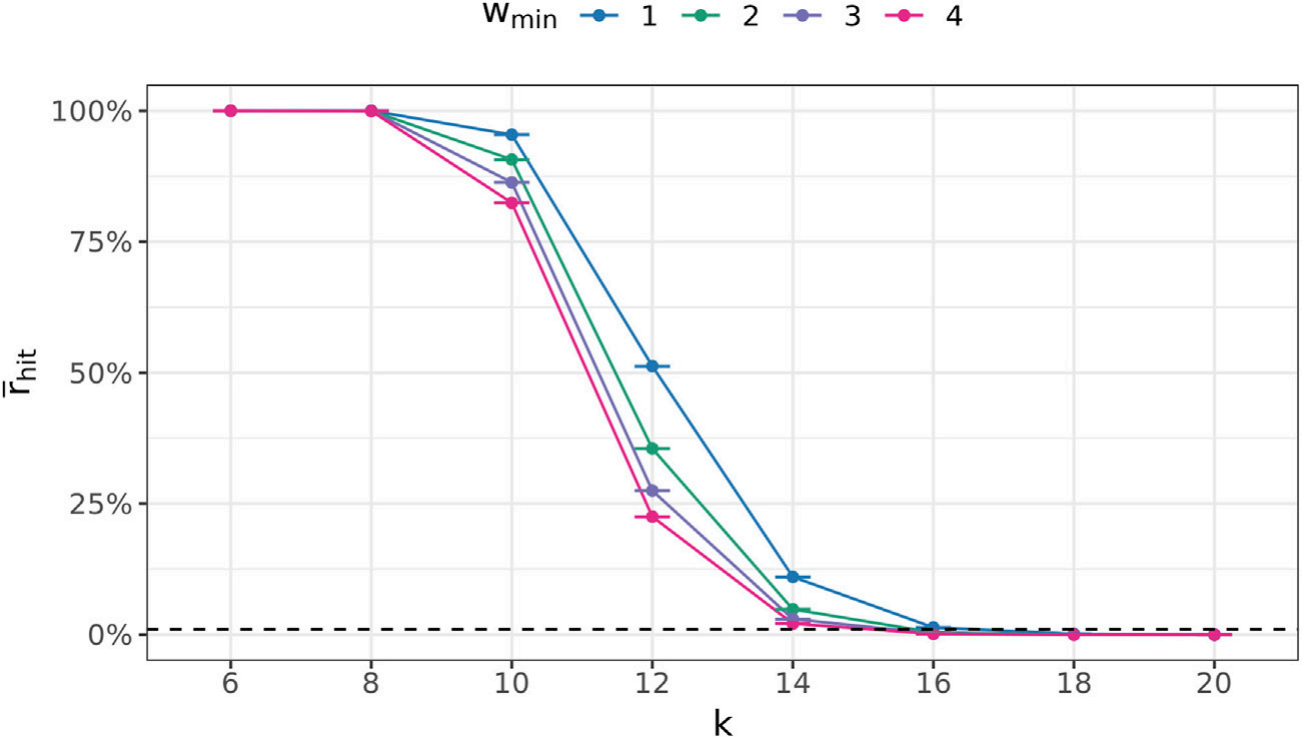
Average fraction ${\bar{r}}_{\text{hit}}$ of random (*k* + 1)-mers that are also contained in the true (*k* + 1)-mer multiset $S_{t}$ at least *w*_min_ times. Error bars are hardly noticeable, indicating small statistical fluctuations.

**FIGURE 8:**
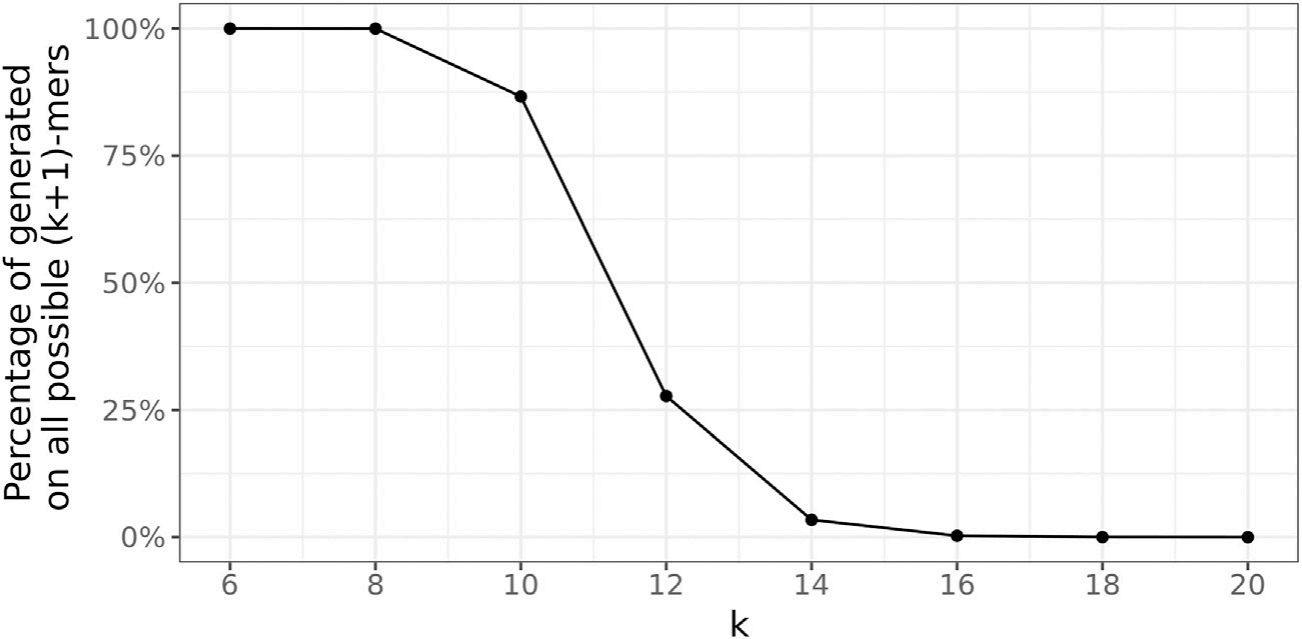
Percentage of unique true (*k* + 1)-mers on all possible 4^*k*+1^ unique (*k* + 1)-mers.

**FIGURE 9:**
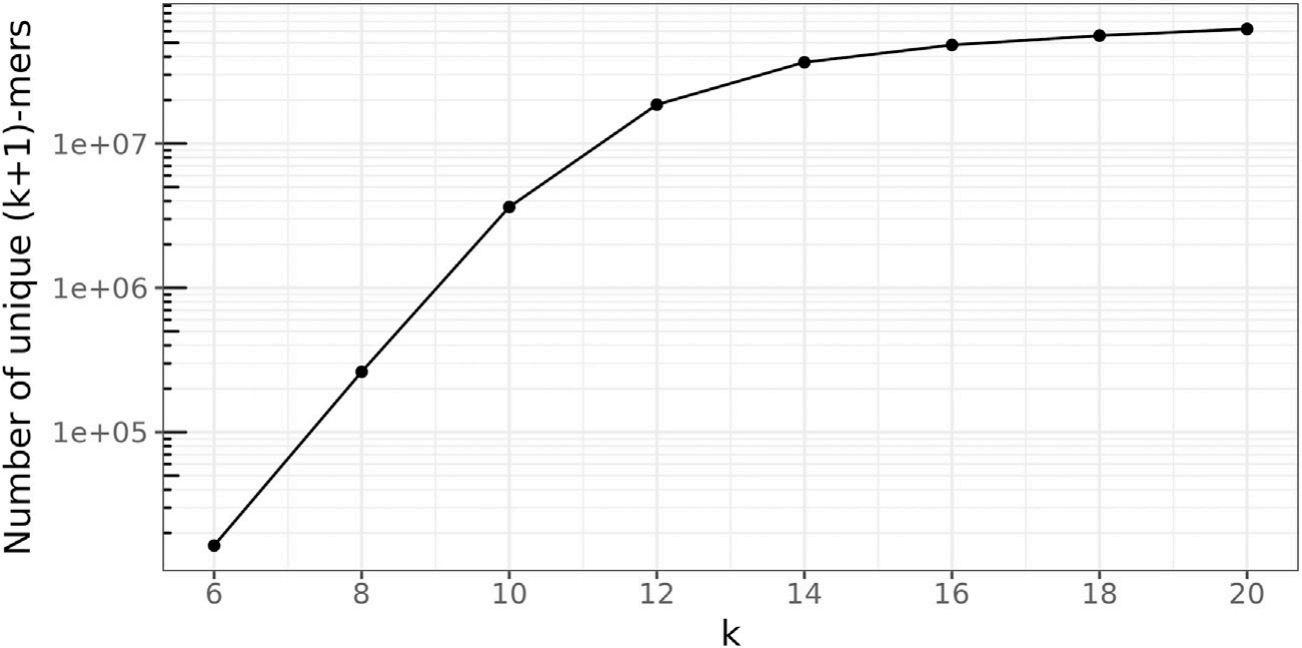
Number of unique true (*k* + 1)-mers on the log scale.

Figure 7 depicts these ratios for all k∈K and *w*_min_ ∈ {1, 2, 3, 4}. For *k* ≤8, each (*k* + 1)-mer in $S_{t}$ is also contained in $S_{r}$ at least four times (i.e., ${\bar{r}}_{\text{hit}}=100\%$ ). Even all 4^*k*+1^ distinct (*k* + 1)-mers that can be generated from an alphabet of nucleotides A, C, T, and G are contained within $S_{t}$ in this case, as shown in Figure 8. This demonstrates that larger *k* values need to be used to achieve that the (*k* + 1)-mers carry at least some meaningful information. However, it is not until *k* = 16 that a significance level of 1% (dashed line) is achieved, at least for *w*_min_ >1 (Figure 7), and hardly any of all 4^*k*+1^ distinct (*k* + 1)-mers occurs in $S_{t}$ (Figure 8). For *k* ≥18, ${\bar{r}}_{\text{hit}}<1\%$ even for *w*_min_ = 1. However, now, more than three-quarters of the (*k* + 1)-mers in $S_{t}$ are unique (see Figure 9). This conceals many sequence similarities between the genomes in the database graph, rendering this choice unsuitable for the presented approach. Thus, *k* = 16 is used instead.

#### 4.2.2 Sequence alignments

For sequence alignments, we applied match costs of 1, mismatch costs of −2, and gap penalties of −2 for opening and extending a gap. The *E*-value threshold was set at 10^–3^. These are common settings, which assume 95% of sequence conservation.

### 4.3 Comparison with MITOS2

Table 1 summarizes the annotation quality of DeGeCI and MITOS2 for all predictions together and also for the different groups of genes: proteins, tRNAs, and rRNAs. RefSeq 89 was used as a reference database for both tools. DeGeCI identified all of the rRNA and protein and 99% of tRNA RefSeq predictions with an equal gene and strand annotation. Thus, DeGeCI obtains an even larger number of correct predictions than MITOS2 (only 99.1% of the protein and 98.7% of the tRNA RefSeq predictions). A genewise and taxonomic breakdown of the results of both tools is compiled in Supplementary Material: Supplementary Tables S1–S11.

**TABLE 1:** Comparison of DeGeCI and MITOS2 predictions with RefSeq 89 annotations. Here, the number of RefSeq predictions $n_{RefSeq}$ , equal predictions (equal), predictions with different strand annotations (Δ±), predictions where gene annotations are different (different), false negatives (FNs), and false positives (FPs) of both tools for each type of gene (protein, tRNA, rRNA, and all) are shown. The percentage of equal DeGeCI/MITOS2 predictions with respect to RefSeq predictions is given in parentheses. Results that have better agreement with RefSeq predictions are highlighted in bold.

	$n_{RefSeq}$		Equal	Δ±	Different	FN	FP
Protein	1,302	DeGeCI	**1,302** (100%)	0	**0**	**0**	**0**
		MITOS2	1,290 (99.1%)	0	17	12	13
tRNA	2,162	DeGeCI	**2,141** (99.0%)	11	**1**	**9**	3
		MITOS2	2,134 (98.7%)	11	4	13	**0**
rRNA	200	DeGeCI	200 (100%)	0	0	0	0
		MITOS2	200 (100%)	0	0	0	0
All	3,664	DeGeCI	**3,643** (99.4%)	11	**1**	**9**	**3**
		MITOS2	3,624 (98.9%)	11	21	25	13

The main cause for the few RefSeq tRNA predictions without equal DeGeCI predictions is the annotation of opposing strands, i.e., corresponding RefSeq and DeGeCI predictions, which are classified as having a strand difference. This affects 11 predictions (see Supplementary Material: Supplementary Table S12 for details). In each such case, more than 95% of DeGeCI positions are shared with RefSeq positions, suggesting a high agreement of both predictions. DeGeCI also always annotates the negative strand, whereas RefSeq annotates the positive strand. Since a special “complement” tag needs to be set for a RefSeq annotation to indicate that the gene is located on the negative strand, there is a reason to presume that this tag was simply forgotten in the corresponding RefSeq entries. The fact that all 11 predictions were also annotated with the opposite strand by MITOS2 supports this hypothesis. Furthermore, nine of the RefSeq predictions stem from the same genome, and not a single gene of this genome is marked with the complement tag, indicating a systematic error in these RefSeq annotations.

There is one DeGeCI prediction that annotates a different gene rather than the corresponding RefSeq prediction, i.e., which has the classification as “different.” It involves the annotation of a different anticodon type of a leucine tRNA. As discussed in Bernt et al. (2013), there are inconsistencies in the naming scheme of RefSeq annotations, resulting in misannotations of the anticodon types of serine and leucine tRNAs (also cf. Supplementary Material: Section 2). Again, MITOS2 identifies the same discrepancy, leaving little doubt that the anticodon type of the RefSeq prediction should be different (cf. Supplementary Material: Supplementary Table S13).

The remaining nine RefSeq predictions without the corresponding equal DeGeCI prediction are FNs (25 in MITOS2), accounting for only 0.2% of the RefSeq entries ( ≈0.7% for MITOS2). Three of them are also not predicted by MITOS2 (i.e., three FNs are shared by both tools), indicating an increased likelihood that they are misannotations in RefSeq.

There are very few (three) DeGeCI annotations that are classified as FPs, all of which are tRNAs. MITOS2, on the other hand, predicts 13 FPs, which are all proteins. For a complete list of FNs of both tools, see Supplementary Material: Supplementary Tables S14, S15 and for their FPs, see Supplementary Table S16.

### 4.4 Accuracy of gene predictions

The majority of the DeGeCI predictions are in much better agreement with the RefSeq predictions than the threshold of 75% overlap requires (cf. Supplementary Material: Supplementary Table S17). More precisely, the average fractions of the DeGeCI positions that are shared with those predicted by RefSeq exceed 98.7%, and the average fractions of RefSeq positions that are shared with those predicted by DeGeCI exceed 97.3% for all types of genes (similar for MITOS2).

DeGeCI also predicts start and end positions with fairly high precision. Table 2 shows that in 96% of the tRNA, 85% of the protein, and 78% of rRNA predictions, the start and stop positions vary by less than 5 nt from the RefSeq annotations. As can be seen from the table, 8% more of DeGeCI′s rRNA predictions were generated with this precision. For proteins and tRNAs, slightly more of the MITOS2 position predictions have a maximum deviation of 5 nt (5% and 3%). One has to keep in mind, however, that MITOS2 also produced more FP and FN predictions for both of these categories (see Table 1). Moreover, for proteins, a possible explanation for this discrepancy could be as follows: after producing protein position predictions, MITOS2 searches the proximity of every start and end position for start and stop codons, respectively. If valid start or stop codons are found, the gene boundary predictions are adapted accordingly. While this approach presumably improves the accuracy of the protein boundary predictions, there are several lineage-specific variations in the standard genetic code for mitogenomes. Thus, to detect appropriate start and end codons for the input genome, the adequate genetic code table must be specified by the user. Since DeGeCI tries to minimize the required amount of knowledge about the input genome and/or user expertise, this is currently not implemented in the DeGeCI pipeline. However, we would like to point out that such an extension would be possible. As soon as slightly larger deviations are considered (i.e., Δ nt ≥10), DeGeCI again produces more protein predictions than MITOS2 (see bold entries in Table 2).

**TABLE 2:** Percentages of corresponding DeGeCI/MITOS2 and RefSeq predictions with start and stop positions of less than Δ nt. The higher rates are highlighted in bold.

Δ nt	Protein [%]		tRNA [%]		rRNA [%]	
	DeGeCI	MITOS2	DeGeCI	MITOS2	DeGeCI	MITOS2
5	85	**90**	96	**99**	**78**	70
10	**94**	92	98	**100**	**80**	79
25	**97**	94	100	100	88	**90**
50	**99**	97	100	100	94	**96**
70	**99**	98	100	100	**100**	98
100	**100**	99	100	100	100	100

### 4.5 RefSeq 204 as a reference database

We also validated DeGeCI′s performance using the more recent RefSeq release RefSeq 204, which contains 9,877 species, for the database graph. Using this larger database, two previous FNs and one FP could be eliminated in the sample set. None of the remaining predictions was impaired (for details, see Supplementary Material: Supplementary Table S18). Since MITOS2 currently only offers prepared databases for RefSeq 39, RefSeq 63, and RefSeq 89, a comparative analysis with MITOS2 could not be carried out for RefSeq 204.

### 4.6 Runtime and scalability

To generate its reference database, MITOS2 needs to retrieve amino acid sequences from the RefSeq release to be used. From these sequences, a new BLAST database needs to be built. Moreover, HMM models need to be generated that use these sequences together with their phylogenetic classification. Lastly, new covariance models need to be built for RNAs, which require manual user interaction. All these steps taken together render database updates a rather tedious task. Contrarily, DeGeCI allows for a fully automated effortless inclusion of additional species to the existing database or the creation of a new database (for details, see Supplementary Material: Section 4), facilitating keeping pace with the increasing amount of available mitochondrial sequence data.

Another important aspect to consider is the scalability of the time requirements for the annotation process with respect to the database size. To compare the impact of the database size on the runtime of both tools, DeGeCI and MITOS2, the sample set was annotated using different RefSeq releases with a different number of species as a reference database. Table 3 shows the average runtimes for the annotation of one input genome on a computer with an AMD Ryzen^TM^ 7 1700 processor with 3 GHz. Since MITOS2 cannot be run in parallel, DeGeCI was run in the single-thread mode for a fair comparison. MITOS2 shows a clear increase in runtime with larger RefSeq releases, whereas DeGeCI is hardly affected by the database growth. DeGeCI also almost always runs considerably faster, e.g., even more than six times as fast as RefSeq 89. The only exception is for RefSeq 39. This is because the small number of species in this RefSeq release results in large unmapped regions in the subgraph, causing comparably long bridging times. DeGeCI runtimes were also measured for RefSeq 204, which includes even more species, showing that the trend of hardly impacted runtimes continues.

**TABLE 3:** Comparison of runtimes. Here, the number $n_{RefSeq}$ of entries in the respective RefSeq release and the average runtime ${\bar{t}}_{\text{DeGeCI}}$ of DeGeCI with one thread and ${\bar{t}}_{\text{MITOS}2}$ of MITOS2 (cannot be run in parallel) are shown. Except for RefSeq 39, DeGeCI runs noticeably faster than MITOS2, and increases in the database size hardly impact the runtime.

	Number of species	${\bar{t}}_{\text{DeGeCI}}$ (one thread) [min]	${\bar{t}}_{\text{MITOS}2}$ [min]	${\bar{t}}_{\text{MITOS}2}/{\bar{t}}_{\text{DeGeCI}}$
RefSeq 39	1,878	4.22	3.77	0.89
RefSeq 63	3,842	2.74	9.11	3.32
RefSeq 89	8,015	2.32	14.40	6.21
RefSeq 204	9,877	2.39	–	–

Nowadays, every modern CPU offers multiple hardware threads. DeGeCI has the advantage that it can (unlike MITOS2) be run in parallel. Preliminary tests on RefSeq 89 with two threads resulted in an average runtime of 1.4 min compared to 2.32 min for one thread. This corresponds to a speed-up value of approximately 1.66 and an efficiency value of approximately 0.83.

## 5 Discussion

This contribution describes a new method, DeGeCI, for an efficient, automatic *de novo* annotation of mitochondrial genomes. The underlying reference database, which comprises a comprehensive collection of mitogenomes, is represented as a richly annotated de Bruijn graph. To generate gene predictions for an input genome sequence, DeGeCI utilizes a clustering technique that is based on the mapping information of this sequence to the graph. DeGeCI produces gene predictions of high conformity with expert-curated annotations of RefSeq, particularly showing the high precision of gene boundaries. Compared to the standard annotation tool MITOS2, DeGeCI generates predictions of at least equal quality, requires less time for annotation when using larger databases, and features better database scalability. Different from MITOS2, the new method, DeGeCI, offers a fully automated update of the reference database and can be run in parallel. Altogether, we could demonstrate that DeGeCI is well suited for large-scale annotations of mitochondrial sequence data.

### 5.1 Limitations

Similar to all database-based annotation approaches (e.g., MITOS, DOGMA, MOSAS, and MitoFish), DeGeCI requires a database containing annotated mitochondrial genome information that includes a certain minimum diversity of species to enable high-quality annotation of mitogenomes across a broad taxonomic spectrum. Otherwise, the annotation quality might be lower, and there might be many and/or relatively large unmapped sequence segments of the input genome. The latter leads to larger annotation times, since a comparably large amount of runtime is required to bridge these unmapped segments in the corresponding subgraph (cf. Section 2.2.2).

DeGeCI has been developed with the prior aim in mind to embed complete genomic sequences of mitochondria. These are generally generated by applying a mixture of long-read and short-read sequencing techniques. Due to the higher error rates associated with long-read sequencing data, using only long-read sequencing data for graph construction might affect the quality of gene predictions obtained using DeGeCI.

### 5.2 Future work

The implementation of a taxonomic filter that would allow the use of only database species of a user-supplied taxonomic classification is our agenda for future work. The use of such a filter could be advantageous with respect to annotation time or quality when a specific taxonomic group of the input sequence is known.

As discussed in Section 4.4, the result accuracy of the DeGeCI protein boundary predictions could likely be improved by scanning the proximity for start and stop codons. Since this requires the user to specify adequate genetic code tables and thus a certain degree of knowledge about the input sequence and/or user expertise, this is currently not part of DeGeCI. This could be implemented in a future version of DeGeCI, allowing the optional refinement of protein predictions.

The focus of this study has been on mitochondrial genomes. An application of the presented methods to nuclear genomes could, hence, be an interesting aspect to be explored in future studies. Suitable parameter settings for *k* could be determined similarly as suggested in this work.

Moreover, the implementation of a public web server version of DeGeCI is planned.

## Data Availability

Publicly available datasets were analyzed in this study. These data can be found at: https://doi.org/10.5281/zenodo.8101631 (RefSeq mitochondrial nucleotide sequences for releases 89 and 204).

## Footnotes

1
https://git.informatik.uni-leipzig.de/lfiedler/degeci

2
https://doi.org/10.5281/zenodo.7010767

## Abbreviations

DeGeCI, De Bruijn graph Gene Cluster Identification; CC, connected component; FN, false negative; FP, false positive; RFD, relative frequency distribution; SGT, single genome trail.
