# Supplementary material for: Fully automated annotation of mitochondrial genomes using a cluster-based approach with de Bruijn graphs
Source: Front Genet. 2023 Aug 10;14:1250907. doi: 10.3389/fgene.2023.1250907 (PMC10448254; doi:10.3389/fgene.2023.1250907)
Supplement: Supplementary file 1 [file DataSheet1.PDF]

Supplementary Material

1 IMPLEMENTATION

DeGeCI employs an indexed PostgreSQL database (Stonebraker, 1987) to achieve permanent storage of the de-Brujin graph. The graph must hence not be constructed for each annotation and does not need to fit into main memory but can still be accessed in a fast manner. The database only stores the edges, i.e., $(k+1)$-mers of *MDBG* as these are sufficient to define the graph.

The proposed methods are completely implemented in Apache Spark (Veith and de Assuncao, 2019), a unified analytics engine for large-scale data processing. This makes the usage of the programs very scalable, as they can be launched on a single computer with one or multiple threads or, if available, be run on a computing cluster. To initiate the annotation of a mitogenome, its nucleotide sequence can either be provided as a file in FASTA format or as plain text.

2 NOMENCLATURE FOR GENE ANNOTATIONS

In our study we followed the gene naming nomenclature suggested by (Boore, 2006). This comprises denoting protein-coding genes based on the human gene nomenclature (Wain et al., 2002) (but in lower case). For ribosomal RNAs, *rrnS* is used to refer to the small and *rrnL* to refer to the large subunit ribosomal RNA. tRNA-encoding genes are denoted with the one-letter code for the associated amino acid, e.g., *Q* for glutamine tRNA. To distinguish between the two types of Serine and Leucine tRNAs respectively, the recognized codon is used, in detail, *S1* for *AGN* or *AGY*, *S2* for *UCN*, *L1* for *CUN*, and *L2* for *UUR*. However, as reported in (Bernt et al., 2013), naming inconsistencies within RefSeq result in some erroneous or incomplete annotations of these tRNAs. The latter thereby refers to the case where the anticodon type is not specified.

3 DETAILS ON GRAPH AND DATABASE STRUCTURE

The subsequences of genomes used for the pairwise sequence alignments cannot be retrieved from $MDBG[\mathcal{K}_{\mathrm{r_{in}}}]$, as they correspond to low mapping (or no mapping) $(k+1)$-mers and were thus never incorporated in the subgraph. One option to retrieve them would be to conduct a graph traversal on *MDBG*, i.e., extracting the $(k+1)$-mers on the associated connecting paths and subsequently assembling them into subsequences. However, this is rather time-consuming. We thus additionally store the complete nucleotide sequence (not its $(k+1)$-mers) of each genome incorporated in *MDBG*. Now this task reduces to simple substring extractions, as we already know all required positions.

4 ADDING GENOMES TO THE GRAPH

Using (k+1)-mers of variable lengths depending on the level of coverage makes the inclusion of additional genomes in the database an extremely complicated and costly task. This is because, generally, this inclusion impacts the coverage of the (k+1)-mers in the graph. The lengths of many (k+1)-mers might hence need to be adapted which not only affects the respective (k+1)-mers itself but also all incident edges, such that large portions of the graph need to be altered. For this purpose, DeGeCI makes use of a fixed value for k and handles low-coverage regions in a second stage.

In DeGeCI, the inclusion of an additional genome r requires only the following few simple steps.

1. The genome sequence must be stored in the genome table of the database.
2. The $(k+1)$-mers of the genome sequence must be generated and added to the database table of already existing edges.
3. The GenBank entry of r must be parsed to conform to the used nomenclature and stored in the gene table of the database.

None of the existing data needs to be modified in any way.

5 GENERAL SETTINGS AND EMPIRICAL DETERMINATION OF STATISTICAL PARAMETERS FOR SEQUENCE ALIGNMENTS

For the sequence alignment, an alignment matrix was used with match costs of 1 and mismatch costs of −2. Moreover, gap penalties of −2 for opening and extending a gap were applied. These settings are used as default settings in BLAST which assume 95% of sequence conservation. To render the quality of the sequence alignments comparable, the *E-values* of the resulting alignment scores are computed. Given an alignment with score S, the E-value is the expected number of alignments with score at least S. This means, the smaller the E-value, the higher the alignment quality. Only alignments with E-value $\leq 10^{-3}$ are retained, as this is an often used cutoff value. The E-value computation involves statistical parameters λ, K, H, which for gapped alignments must be inferred from simulated sequences. To this end we aligned a large number of random sequence tuples and recorded their best scores S' and alignment lengths l'. To generate sequence tuples of representative sequence composition, we once generated long random sequences (by concatenating and shuffling the sequences used for constructing *MDBG*) and split them into sequence chunks of average genome length. The maximum-likelihood method was then applied to estimate the desired parameters by assuming an extreme value distribution of S', as suggested by (Altschul and Gish, 1996; Lawless, 2011).

6 LIST OF MITOGENOMES USED FOR THE EVALUATION (BY NCBI ACCESSION)

NC_008667, NC_008682, NC_010341, NC_013616, NC_013994, NC_013995, NC_018035, NC_018036, NC_018037, NC_018038, NC_018039, NC_018134, NC_018366, NC_021414, NC_021418, NC_023534, NC_024277, NC_024422, NC_024623, NC_024645, NC_025590, NC_025770, NC_026118, NC_026458, NC_026870, NC_027069, NC_028172, NC_028173, NC_028224, NC_031808, NC_036033, NC_036034, NC_014053, NC_016724, NC_017744, NC_019622, NC_022185, NC_022689, NC_023118, NC_023791, NC_024398, NC_024399, NC_024403, NC_024410, NC_024416, NC_024417, NC_024727, NC_025764, NC_026840, NC_028207, NC_030370, NC_033540, NC_034232, NC_034233, NC_034663, NC_034664, NC_034754, NC_036057, NC_037397, NC_012706, NC_013834, NC_013996, NC_016178, NC_016707, NC_018358, NC_018595, NC_020622, NC_020623, NC_020686, NC_020694, NC_020704, NC_020736, NC_020745, NC_031835, NC_018811, NC_036397, NC_036398, NC_036399, NC_036400, NC_036401, NC_036402, NC_036403, NC_036404, NC_036405, NC_036406, NC_036407, NC_036408, NC_007896, NC_017749, NC_022693, NC_028731, NC_029373, NC_037604, NC_037887, NC_017871, NC_027421, NC_027505, NC_029231, NC_022827, NC_022828

7 SUPPLEMENTARY TABLES AND FIGURES

7.1 Tables

Table SS1. Comparison of the DeGeCI (gray) and MITOS2 (white) predictions with the RefSeq89 annotations for proteins. Shown are the number of RefSeq predictions $n_{\texttt{RefSeq}}$, equal predictions (equal), predictions with different strand annotations (Δ±), predictions where the gene annotations are different (different), false negatives (FN), and false positives (FP) of both tools. The fraction of equal DeGeCI/MITOS2 predictions with respect to the RefSeq predictions is given in parentheses.

		$n_{\texttt{RefSeq}}$	equal	Δ±	different	FN	FP
atp6	DeGeCI	100	100 (1.000)	0	0	0	0
atp6	MITOS2	100	100 (1.000)	0	0	0	0
atp8	DeGeCI	100	100 (1.000)	0	0	0	0
atp8	MITOS2	100	100 (1.000)	0	0	0	0
cob	DeGeCI	100	100 (1.000)	0	0	0	0
cob	MITOS2	100	100 (1.000)	0	0	0	6
cox1	DeGeCI	100	100 (1.000)	0	0	0	0
cox1	MITOS2	100	100 (1.000)	0	0	0	0
cox2	DeGeCI	100	100 (1.000)	0	0	0	0
cox2	MITOS2	100	100 (1.000)	0	0	0	0
cox3	DeGeCI	100	100 (1.000)	0	0	0	0
cox3	MITOS2	100	100 (1.000)	0	0	0	0
nad1	DeGeCI	100	100 (1.000)	0	0	0	0
nad1	MITOS2	100	100 (1.000)	0	0	0	0
nad2	DeGeCI	100	100 (1.000)	0	0	0	0
nad2	MITOS2	100	100 (1.000)	0	0	0	0
nad3	DeGeCI	100	100 (1.000)	0	0	0	0
nad3	MITOS2	100	100 (1.000)	0	0	0	0
nad4	DeGeCI	100	100 (1.000)	0	0	0	0
nad4	MITOS2	100	100 (1.000)	0	1	0	1
nad4l	DeGeCI	100	100 (1.000)	0	0	0	0
nad4l	MITOS2	100	88 (0.880)	0	0	12	1
nad5	DeGeCI	102	102 (1.000)	0	0	0	0
nad5	MITOS2	102	102 (1.000)	0	13	0	5
nad6	DeGeCI	100	100 (1.000)	0	0	0	0
nad6	MITOS2	100	100 (1.000)	0	0	0	0
all proteins	DeGeCI	1302	1302 (1.000)	0	0	0	0
all proteins	MITOS2	1302	1290 (0.991)	0	17	12	13

Table SS2. Comparison of the DeGeCI (gray) and MITOS2 (white) predictions with the RefSeq89 annotations for rRNA genes. Shown are the number of RefSeq predictions $n_{\texttt{RefSeq}}$, equal predictions (equal), predictions with different strand annotations (Δ±), predictions where the gene annotations are different (different), false negatives (FN), and false positives (FP) of both tools. The fraction of equal DeGeCI/MITOS2 predictions with respect to the RefSeq predictions is given in parentheses.

		$n_{\texttt{RefSeq}}$	equal	Δ±	different	FN	FP
rrnL	DeGeCI	100	100 (1.000)	0	0	0	0
rrnL	MITOS2	100	100 (1.000)	0	0	0	0
rrnS	DeGeCI	100	100 (1.000)	0	0	0	0
rrnS	MITOS2	100	100 (1.000)	0	0	0	0
all rRNAs	DeGeCI	200	200 (1.000)	0	0	0	0
all rRNAs	MITOS2	200	200 (1.000)	0	0	0	0

Table SS3. Comparison of the `DeGeCI` (gray) and `MITOS2` (white) predictions with the `RefSeq89` annotations for tRNAs. Shown are the number of `RefSeq` predictions $n_{\texttt{RefSeq}}$, equal predictions (equal), predictions with different strand annotations (Δ±), predictions where the gene annotations are different (different), false negatives (FN), and false positives (FP) of both tools. The fraction of equal `DeGeCI`/`MITOS2` predictions with respect to the `RefSeq` predictions is given in parentheses.

		$n_{\texttt{RefSeq}}$	equal	Δ±	different	FN	FP
A	DeGeCI	98	98 (1.000)	0	0	0	0
A	MITOS2	98	98 (1.000)	0	0	0	0
C	DeGeCI	98	97 (0.990)	1	0	0	0
C	MITOS2	98	97 (0.990)	1	0	0	0
D	DeGeCI	98	96 (0.980)	0	0	2	1
D	MITOS2	98	98 (1.000)	0	0	0	0
E	DeGeCI	98	95 (0.969)	2	0	1	0
E	MITOS2	98	96 (0.980)	2	0	0	0
F	DeGeCI	97	96 (0.990)	0	0	1	1
F	MITOS2	97	92 (0.948)	0	0	5	0
G	DeGeCI	98	97 (0.990)	1	0	0	0
G	MITOS2	98	97 (0.990)	1	0	0	0
H	DeGeCI	98	98 (1.000)	0	0	0	0
H	MITOS2	98	92 (0.939)	0	0	6	0
I	DeGeCI	98	98 (1.000)	0	0	0	0
I	MITOS2	98	97 (0.990)	0	0	0	0
K	DeGeCI	98	98 (1.000)	0	0	0	0
K	MITOS2	98	98 (1.000)	0	0	0	0
L1	DeGeCI	98	95 (0.969)	0	1	2	0
L1	MITOS2	98	97 (0.990)	0	1	0	0
L2	DeGeCI	98	98 (1.000)	0	0	0	1
L2	MITOS2	98	98 (1.000)	0	1	0	0
M	DeGeCI	100	99 (0.990)	1	0	0	0
M	MITOS2	100	99 (0.990)	1	0	0	0
N	DeGeCI	98	98 (1.000)	0	0	0	0
N	MITOS2	98	98 (1.000)	0	0	0	0
P	DeGeCI	98	96 (0.980)	2	0	0	0
P	MITOS2	98	96 (0.980)	2	0	0	0
Q	DeGeCI	98	97 (0.990)	1	0	0	0
Q	MITOS2	98	97 (0.990)	1	0	0	0
R	DeGeCI	98	98 (1.000)	0	0	0	0
R	MITOS2	98	98 (1.000)	0	1	0	0
S1	DeGeCI	64	98 (1.531)	0	0	0	0
S1	MITOS2	64	98 (1.531)	0	0	0	0
S2	DeGeCI	65	98 (1.508)	0	0	0	0
S2	MITOS2	65	97 (1.492)	0	0	0	0
T	DeGeCI	101	98 (0.970)	1	0	2	0
T	MITOS2	101	98 (0.970)	1	1	2	0
V	DeGeCI	98	98 (1.000)	0	0	0	0
V	MITOS2	98	98 (1.000)	0	0	0	0
W	DeGeCI	100	98 (0.980)	1	0	1	0
W	MITOS2	100	98 (0.980)	1	0	0	0
Y	DeGeCI	98	97 (0.990)	1	0	0	0
Y	MITOS2	98	97 (0.990)	1	0	0	0
all tRNAs	DeGeCI	2162	2141 (0.990)	11	1	9	3
all tRNAs	MITOS2	2162	2134 (0.987)	11	4	13	0

Table SS4. Comparison of the `DeGeCI` (gray) and `MITOS2` (white) predictions with the `RefSeq89` annotations of taxonomic group Actinopterygii. Shown are the number of `RefSeq` predictions $n_{\texttt{RefSeq}}$, equal predictions (equal), predictions with different strand annotations (Δ±), predictions where the gene annotations are different (different), false negatives (FN), and false positives (FP) of both tools. The fraction of equal `DeGeCI`/`MITOS2` predictions with respect to the `RefSeq` predictions is given in parentheses.

		$n_{\texttt{RefSeq}}$	equal	Δ±	different	FN	FP
protein	DeGeCI	416	416 (1.000)	0	0	0	0
protein	MITOS2	416	404 (0.971)	0	12	12	0
tRNA	DeGeCI	704	703 (0.999)	1	0	0	0
tRNA	MITOS2	704	701 (0.996)	1	2	0	0
rRNA	DeGeCI	64	64 (1.000)	0	0	0	0
rRNA	MITOS2	64	64 (1.000)	0	0	0	0
all genes	DeGeCI	1184	1183 (0.999)	1	0	0	0
all genes	MITOS2	1184	1169 (0.987)	1	14	12	0

Table SS5. Comparison of the `DeGeCI` (gray) and `MITOS2` (white) predictions with the `RefSeq89` annotations of taxonomic group Amphibia. Shown are the number of `RefSeq` predictions $n_{\texttt{RefSeq}}$, equal predictions (equal), predictions with different strand annotations (Δ±), predictions where the gene annotations are different (different), false negatives (FN), and false positives (FP) of both tools. The fraction of equal `DeGeCI`/`MITOS2` predictions with respect to the `RefSeq` predictions is given in parentheses.

		$n_{\texttt{RefSeq}}$	equal	Δ±	different	FN	FP
protein	DeGeCI	52	52 (1.000)	0	0	0	0
protein	MITOS2	52	52 (1.000)	0	0	0	6
tRNA	DeGeCI	91	88 (0.967)	1	0	2	0
tRNA	MITOS2	91	88 (0.967)	1	0	2	0
rRNA	DeGeCI	8	8 (1.000)	0	0	0	0
rRNA	MITOS2	8	8 (1.000)	0	0	0	0
all genes	DeGeCI	151	148 (0.980)	1	0	2	0
all genes	MITOS2	151	148 (0.980)	1	0	2	6

Table SS6. Comparison of the `DeGeCI` (gray) and `MITOS2`(white) predictions with the `RefSeq89` annotations of taxonomic group Arthropoda. Shown are the number of `RefSeq` predictions $n_{\texttt{RefSeq}}$, equal predictions (equal), predictions with different strand annotations (Δ±), predictions where the gene annotations are different (different), false negatives (FN), and false positives (FP) of both tools. The fraction of equal `DeGeCI`/`MITOS2` predictions with respect to the `RefSeq` predictions is given in parentheses.

		$n_{\texttt{RefSeq}}$	equal	Δ±	different	FN	FP
protein	DeGeCI	351	351 (1.000)	0	0	0	0
protein	MITOS2	351	351 (1.000)	0	2	0	2
tRNA	DeGeCI	594	588 (0.990)	1	1	4	1
tRNA	MITOS2	594	584 (0.983)	1	1	8	0
rRNA	DeGeCI	54	54 (1.000)	0	0	0	0
rRNA	MITOS2	54	54 (1.000)	0	0	0	0
all genes	DeGeCI	999	994 (0.995)	1	1	4	1
all genes	MITOS2	999	990 (0.991)	1	3	8	2

Table SS7. Comparison of the `DeGeCI` (gray) and `MITOS2` (white) predictions with the `RefSeq89` annotations of taxonomic group Mammalia. Shown are the number of `RefSeq` predictions $n_{\texttt{RefSeq}}$, equal predictions (equal), predictions with different strand annotations (Δ±), predictions where the gene annotations are different (different), false negatives (FN), and false positives (FP) of both tools. The fraction of equal `DeGeCI`/`MITOS2` predictions with respect to the `RefSeq` predictions is given in parentheses.

		$n_{\texttt{RefSeq}}$	equal	Δ±	different	FN	FP
protein	DeGeCI	195	195 (1.000)	0	0	0	0
protein	MITOS2	195	195 (1.000)	0	0	0	4
tRNA	DeGeCI	330	330 (1.000)	0	0	0	0
tRNA	MITOS2	330	330 (1.000)	0	0	0	0
rRNA	DeGeCI	30	30 (1.000)	0	0	0	0
rRNA	MITOS2	30	30 (1.000)	0	0	0	0
all genes	DeGeCI	555	555 (1.000)	0	0	0	0
all genes	MITOS2	555	555 (1.000)	0	0	0	4

Table SS8. Comparison of the `DeGeCI` (gray) and `MITOS2` (white) predictions with the `RefSeq89` annotations of the non-bilaterian species. Shown are the number of `RefSeq` predictions $n_{\texttt{RefSeq}}$, equal predictions (equal), predictions with different strand annotations (Δ±), predictions where the gene annotations are different (different), false negatives (FN), and false positives (FP) of both tools. The fraction of equal `DeGeCI`/`MITOS2` predictions with respect to the `RefSeq` predictions is given in parentheses.

		$n_{\texttt{RefSeq}}$	equal	Δ±	different	FN	FP
protein	DeGeCI	28	28 (1.000)	0	0	0	0
protein	MITOS2	28	28 (1.000)	0	0	0	0
tRNA	DeGeCI	4	4 (1.000)	0	0	0	0
tRNA	MITOS2	4	4 (1.000)	0	0	0	0
rRNA	DeGeCI	4	4 (1.000)	0	0	0	0
rRNA	MITOS2	4	4 (1.000)	0	0	0	0
all genes	DeGeCI	36	36 (1.000)	0	0	0	0
all genes	MITOS2	36	36 (1.000)	0	0	0	0

Table SS9. Comparison of the `DeGeCI` (gray) and `MITOS2` (white) predictions with the `RefSeq89` annotations of taxonomic group Sauropsida. Shown are the number of `RefSeq` predictions $n_{\texttt{RefSeq}}$, equal predictions (equal), predictions with different strand annotations (Δ±), predictions where the gene annotations are different (different), false negatives (FN), and false positives (FP) of both tools. The fraction of equal `DeGeCI`/`MITOS2` predictions with respect to the `RefSeq` predictions is given in parentheses.

		$n_{\texttt{RefSeq}}$	equal	Δ±	different	FN	FP
protein	DeGeCI	169	169 (1.000)	0	0	0	0
protein	MITOS2	169	169 (1.000)	0	0	0	0
tRNA	DeGeCI	286	286 (1.000)	0	0	0	0
tRNA	MITOS2	286	286 (1.000)	0	0	0	0
rRNA	DeGeCI	26	26 (1.000)	0	0	0	0
rRNA	MITOS2	26	26 (1.000)	0	0	0	0
all genes	DeGeCI	481	481 (1.000)	0	0	0	0
all genes	MITOS2	481	481 (1.000)	0	0	0	0

Table SS10. Comparison of the `DeGeCI` (gray) and `MITOS2` (white) predictions with the `RefSeq89` annotations of taxonomic group Spiralia. Shown are the number of `RefSeq` predictions $n_{\texttt{RefSeq}}$, equal predictions (equal), predictions with different strand annotations (Δ±), predictions where the gene annotations are different (different), false negatives (FN), and false positives (FP) of both tools. The fraction of equal `DeGeCI`/`MITOS2` predictions with respect to the `RefSeq` predictions is given in parentheses.

		$n_{\texttt{RefSeq}}$	equal	Δ±	different	FN	FP
protein	DeGeCI	91	91 (1.000)	0	0	0	0
protein	MITOS2	91	91 (1.000)	0	3	0	1
tRNA	DeGeCI	153	142 (0.928)	8	0	3	2
tRNA	MITOS2	153	141 (0.922)	8	1	3	0
rRNA	DeGeCI	14	14 (1.000)	0	0	0	0
rRNA	MITOS2	14	14 (1.000)	0	0	0	0
all genes	DeGeCI	258	247 (0.957)	8	0	3	2
all genes	MITOS2	258	246 (0.953)	8	4	3	1

Table SS11. Comparison of the `DeGeCI` (gray) and `MITOS2` (white) predictions with the `RefSeq89` annotations considering all taxonomic groups. Shown are the number of `RefSeq` predictions $n_{\texttt{RefSeq}}$, equal predictions (equal), predictions with different strand annotations (Δ±), predictions where the gene annotations are different (different), false negatives (FN), and false positives (FP) of both tools. The fraction of equal `DeGeCI`/`MITOS2` predictions with respect to the `RefSeq` predictions is given in parentheses.

		$n_{\texttt{RefSeq}}$	equal	Δ±	different	FN	FP
protein	DeGeCI	1302	1302 (1.000)	0	0	0	0
protein	MITOS2	1302	1290 (0.991)	0	17	12	13
tRNA	DeGeCI	2162	2141 (0.990)	11	1	9	3
tRNA	MITOS2	2162	2134 (0.987)	11	4	13	0
rRNA	DeGeCI	200	200 (1.000)	0	0	0	0
rRNA	MITOS2	200	200 (1.000)	0	0	0	0
all genes	DeGeCI	3664	3643 (0.994)	11	1	9	3
all genes	MITOS2	3664	3624 (0.989)	11	21	25	13

Table SS12. Genes with positive strand RefSeq89 annotations but negative strand MITOS2 and DeGeCI annotations. A tag needs to be set in the RefSeq GenBank file to annotate that a gene is located on the negative strand. The fact that all predictions of the gene location are on the opposing strand in both tools suggests that this tag was forgotten in the RefSeq annotations.

accession ID		gene	start	end	length	start RefSeq	end RefSeq	length RefSeq
NC_017871	DeGeCI	*P*	16292	16361	70	16291	16362	72
	MITOS2	*P*	16291	16362	72	16291	16362	72
NC_021418	DeGeCI	*E*	14329	14396	68	14329	14397	69
	MITOS2	*E*	14329	14397	69	14329	14397	69
NC_029373	DeGeCI	*C*	3351	3414	64	3351	3414	64
	MITOS2	*C*	3351	3414	64	3351	3414	64
	DeGeCI	*E*	3617	3681	65	3616	3682	67
	MITOS2	*E*	3616	3682	67	3616	3682	67
	DeGeCI	*G*	3545	3614	70	3548	3615	68
	MITOS2	*G*	3548	3615	68	3548	3615	68
	DeGeCI	*M*	3211	3274	64	3209	3276	68
	MITOS2	*M*	3210	3275	66	3209	3276	68
	DeGeCI	*Q*	3480	3543	64	3478	3544	67
	MITOS2	*Q*	3479	3543	65	3478	3544	67
	DeGeCI	*T*	8962	9004	43	8940	9005	66
	MITOS2	*T*	8940	9005	66	8940	9005	66
	DeGeCI	*W*	3415	3480	66	3413	3480	68
	MITOS2	*W*	3415	3480	66	3413	3480	68
	DeGeCI	*Y*	3285	3350	66	3284	3350	67
	MITOS2	*Y*	3285	3349	65	3284	3350	67
	DeGeCI	*P*	9852	9915	64	9851	9916	66
	MITOS2	*P*	9851	9916	66	9851	9916	66

Table SS13: (A) MITOS2 annotation of genes with different annotation in RefSeq89. RefSeq predictions for which there is a second accepted MITOS2 prediction (i.e., with at least 75% of the MITOS2 positions shared with the RefSeq positions and equal gene annotation) are marked with a tick. In all of the ticked predictions, a large number of the MITOS2 positions is shared with the RefSeq positions (overlap MITOS2), but the fraction of RefSeq positions that are shared with the MITOS2 positions (overlap RefSeq) is small, i.e., less than 15%. (B) DeGeCI annotation of genes with different annotation in RefSeq89.

(A)

accession ID	gene	start	end	length	RefSeq gene	RefSeq start	RefSeq end	RefSeq length	overlap MITOS2	overlap RefSeq	other accepted prediction
NC_017749	*lagli*	6084	6239	156	*nad1*	5275	6212	938	0.83	0.14	✓
NC_022693	*lagli*	6092	6235	144	*nad1*	5284	6220	937	0.90	0.14	✓
NC_028731	*lagli*	6084	6239	156	*nad1*	5275	6212	938	0.83	0.14	✓
NC_030370	*nad4*	8165	8173	9	*nad4*	8166	9504	1339	0.89	0.01	✓
NC_036034	*nad5*	12600	12659	60	*nad4*	11320	12701	1382	1.00	0.04	✓
NC_017744	*nad5*	9457	9564	108	*nad4*	8222	9560	1339	0.96	0.08	✓
NC_010341	*nad5*	12600	12659	60	*nad4*	11320	12701	1382	1.00	0.04	✓
NC_013994	*nad5*	12600	12659	60	*nad4*	11320	12701	1382	1.00	0.04	✓
NC_013995	*nad5*	12600	12659	60	*nad4*	11320	12701	1382	1.00	0.04	✓
NC_028224	*nad5*	12600	12659	60	*nad4*	11320	12701	1382	1.00	0.04	✓
NC_036033	*nad5*	12600	12659	60	*nad4*	11320	12701	1382	1.00	0.04	✓
NC_028172	*nad5*	12598	12657	60	*nad4*	11318	12699	1382	1.00	0.04	✓
NC_028173	*nad5*	12600	12659	60	*nad4*	11320	12701	1382	1.00	0.04	✓
NC_026118	*nad5*	11663	11722	60	*nad4*	10383	11764	1382	1.00	0.04	✓
NC_026458	*nad5*	11661	11720	60	*nad4*	10381	11762	1382	1.00	0.04	✓
NC_023534	*nad5*	12600	12659	60	*nad4*	11320	12701	1382	1.00	0.04	✓
NC_024422	*nad5*	11661	11720	60	*nad4*	10381	11762	1382	1.00	0.04	✓
NC_022185	*L1*	12748	12815	68	L2	12748	12815	68	100.00	100.00	
NC_037604	*L2*	8878	8942	65	S2	8874	8942	69	100.00	94.20	
NC_031808	*R*	5088	5158	71	W	5088	5158	71	100.00	100.00	
NC_010341	*T*	4789	4860	72	I	4789	4860	72	100.00	100.00	

(B)

accession ID	gene	start	end	length	RefSeq gene	RefSeq start	RefSeq end	RefSeq length	overlap DeGeCI	overlap RefSeq
NC_022185	*L1*	12749	12779	31	L2	12748	12815	68	100.00	45.59

Table SS14. `MITOS2` false negatives. False negatives found with both `MITOS2` and `DeGeCI` are highlighted in bold.

accession ID	taxonomic group	gene	start	end	length	details
NC_024417	Arthropoda	*F*	6299	6367	69	
**NC_034663**	**Arthropoda**	***F***	**6259**	**6313**	**55**	
NC_017749	Spiralia	*F*	5210	5275	66	
NC_022693	Spiralia	*F*	5218	5283	66	
NC_028731	Spiralia	*F*	5210	5275	66	
NC_016724	Arthropoda	*H*	8057	8121	65	
NC_017744	Arthropoda	*H*	8154	8221	68	
NC_022185	Arthropoda	*H*	8153	8220	68	
NC_022689	Arthropoda	*H*	8144	8210	67	
NC_034232	Arthropoda	*H*	8127	8194	68	
NC_034754	Arthropoda	*H*	8073	8140	68	
NC_010341	Actinopterygii	*nad4*	11030	11326	297	
NC_013994	Actinopterygii	*nad4l*	11030	11326	297	
NC_013995	Actinopterygii	*nad4l*	11030	11326	297	
NC_023534	Actinopterygii	*nad4l*	11030	11326	297	
NC_024422	Actinopterygii	*nad4l*	10091	10387	297	
NC_026118	Actinopterygii	*nad4l*	10093	10389	297	
NC_026458	Actinopterygii	*nad4l*	10091	10387	297	
NC_028172	Actinopterygii	*nad4l*	11028	11324	297	
NC_028173	Actinopterygii	*nad4l*	11030	11326	297	
NC_028224	Actinopterygii	*nad4l*	11030	11326	297	
NC_036033	Actinopterygii	*nad4l*	11030	11326	297	
NC_036034	Actinopterygii	*nad4l*	11030	11326	297	
**NC_027505**	**Amphibia**	***T***	**15755**	**15762**	**8**	second copy of this gene, marked as non-processed pseudogene
**NC_029231**	**Amphibia**	***T***	**15743**	**15806**	**64**	second copy of this gene, marked as non-processed pseudogene

Table SS15. `DeGeCI` false negatives. False negatives found with both `MITOS2` and `DeGeCI` are highlighted in bold.

accession ID	taxonomic group	gene	start	end	length	details
NC_022185	Arthropoda	*D*	3860	3930	71	
NC_023118	Arthropoda	*D*	3831	3897	67	
NC_007896	Spiralia	*E*	14891	14964	74	
**NC_034663**	**Arthropoda**	***F***	**6259**	**6313**	**55**	
NC_024417	Arthropoda	*L1*	12634	12701	68	
NC_022693	Spiralia	*L1*	6294	6362	69	
**NC_027505**	**Amphibia**	***T***	**15755**	**15762**	**8**	second copy of this gene,marked as non-processed pseudogene
**NC_029231**	**Amphibia**	***T***	**15743**	**15806**	**64**	second copy of this gene,marked as non-processed pseudogene
NC_007896	Spiralia	*W*	14687	14752	66	

Table SS16. False positives of `MITOS2` and `DeGeCI`.

	gene	occurances
`DeGeCI`	*D*	1
	F	1
	L2	1
`MITOS2`	*nad4*	1
	nad4l	1
	nad5	5
	cob	6

Table SS17. Mean an median of the fraction of `DeGeCI/MITOS2` positions shared with those predicted by `RefSeq` (column 3-4) and mean and median of the fraction of `RefSeq` positions shared with those predicted by `DeGeCI/MITOS2` (column 5-6).

		DeGeCI/ MITOS2 [%]		RefSeq [%]	
		mean	median	mean	median
protein	DeGeCI	99.52	100.00	99.38	99.73
	MITOS2	98.37	100.00	99.45	100.00
rrna	DeGeCI	98.74	99.82	99.47	99.94
	MITOS2	99.57	99.90	98.89	99.81
trna	DeGeCI	98.91	100.00	97.25	98.51
	MITOS2	99.80	100.00	99.75	100.00

Table SS18. Quality of the `DeGeCI` predictions for `RefSeq204`. Shown are the number of `RefSeq` predictions $n_{\texttt{RefSeq}}$, equal predictions (equal), predictions with different strand annotations (Δ±), predictions where the gene annotations are different (different), false negatives (FN), and false positives (FP) for each type of gene (protein, tRNA, rRNA, all). The percentage of equal `DeGeCI` predictions with respect to the `RefSeq` predictions is given in parentheses. Improvements with respect to using `RefSeq89` as reference database are highlighted in bold (none of the predictions was impaired).

$n_{\texttt{RefSeq}}$		equal	Δ±	different	FN	FP
protein	1302	1302 (100.0%)	0	0	0	0
tRNA	2162	**2143 (99.1%)**	11	1	**7**	**2**
rRNA	200	200 (100%)	0	0	0	0
all genes	3664	**3645(99.5%)**	11	1	**7**	**2**

7.2 Figures

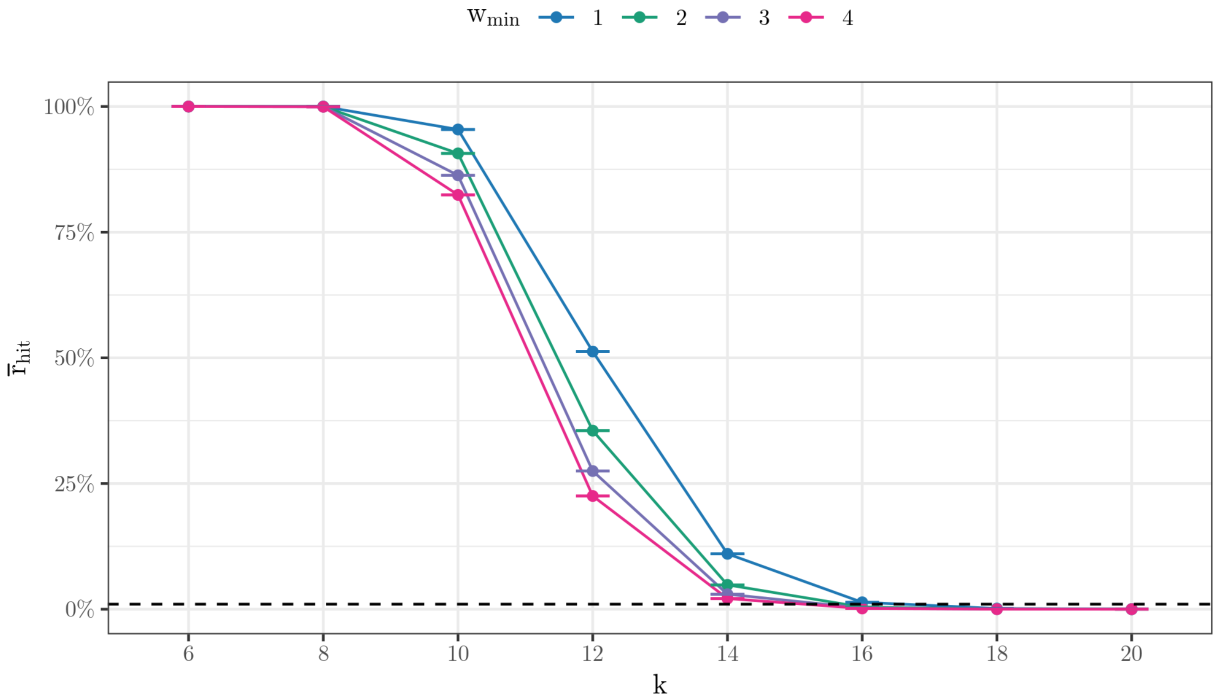

Figure SS1: Average fraction $\overline{r}_{hit}$ of random (k+1)-mers that are also contained in the true (k+1)-mermultiset $\mathcal{S}_t$ at least w_{min} times. Data points are connected by lines to guide the eye. Error bars are hardly noticeable, indicating small statistical fluctuations.

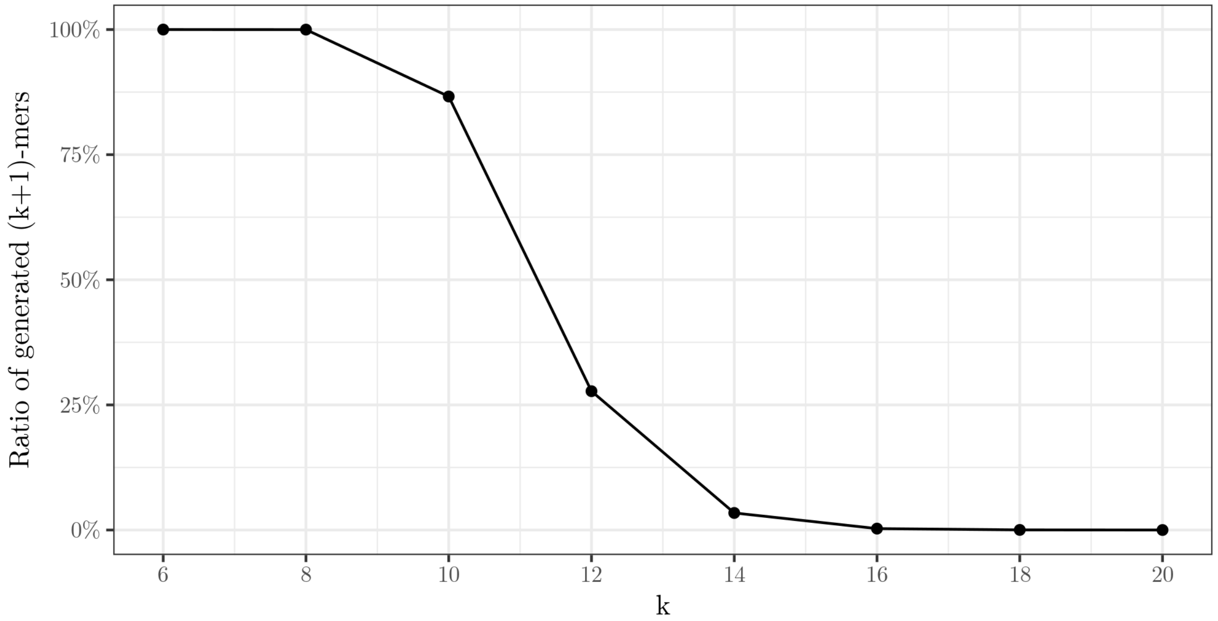

Figure SS2: Percentage of unique true $(k+1)$-mers on all possible 4^{k+1} unique $(k+1)$-mers.

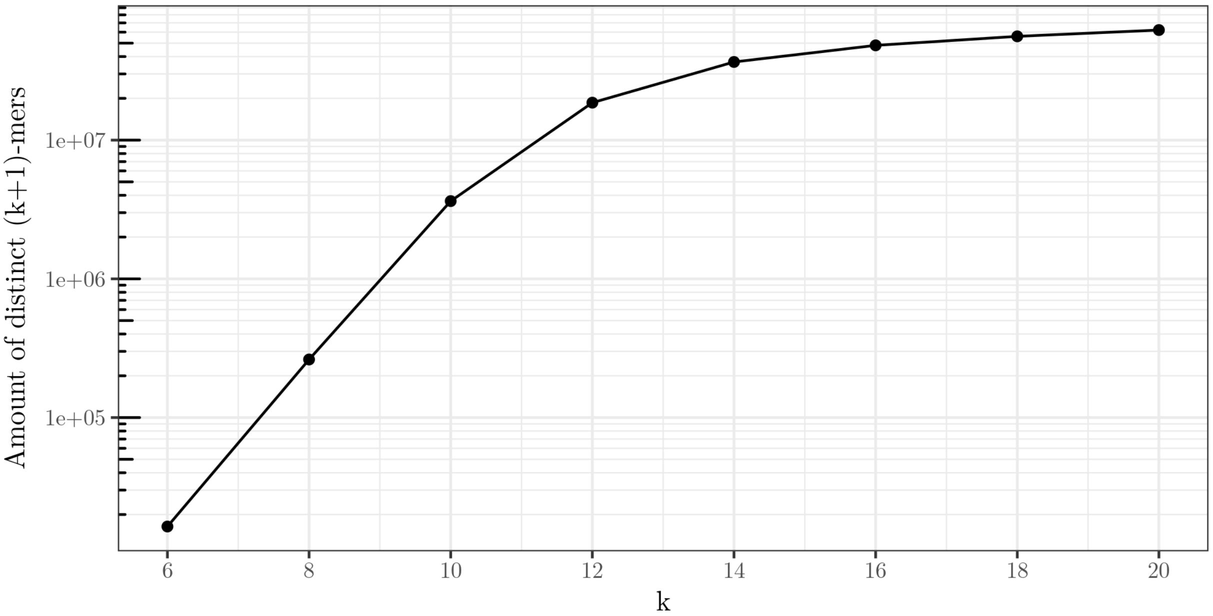

Figure SS3: Number of unique true $(k+1)$-mers in log-scale.

REFERENCES

Altschul, S. F. and Gish, W. (1996). Local alignment statistics (Academic Press), vol. 266 of *Computer Methods for Macromolecular Sequence Analysis*. 460–480. doi:10.1016/S0076-6879(96)66029-7

Bernt, M., Donath, A., Jühling, F., Externbrink, F., Florentz, C., Fritzsch, G., et al. (2013). MITOS: Improved de novo metazoan mitochondrial genome annotation. *Molecular Phylogenetics and Evolution* 69, 313–319. doi:10.1016/j.ympev.2012.08.023

Boore, J. L. (2006). Requirements and Standards for Organelle Genome Databases. *OMICS: A Journal of Integrative Biology* 10, 119–126. doi:10.1089/omi.2006.10.119

Lawless, J. F. (2011). *Statistical Models and Methods for Lifetime Data* (John Wiley & Sons)

Stonebraker, M. (1987). *The design of the Postgres storage system*. Tech. rep., California Univ Berkeley Electronics Research Lab California

Veith, A. d. S. and de Assuncao, M. D. (2019). Apache Spark (Cham: Springer International Publishing). 77–81. doi:10.1007/978-3-319-77525-8_37

Wain, H. M., Bruford, E. A., Lovering, R. C., Lush, M. J., Wright, M. W., and Povey, S. (2002). Guidelines for human gene nomenclature. *Genomics* 79, 464–470. doi:10.1006/geno.2002.6748
